# Precursors of exhausted T cells are pre-emptively formed in acute infection

**DOI:** 10.1038/s41586-024-08451-4

**Published:** 2025-01-08

**Authors:** Talyn Chu, Ming Wu, Barbara Hoellbacher, Gustavo P. de Almeida, Christine Wurmser, Jacqueline Berner, Lara V. Donhauser, Ann-Katrin Gerullis, Siran Lin, J. Diego Cepeda-Mayorga, Iman I. Kilb, Lukas Bongers, Fabio Toppeta, Philipp Strobl, Ben Youngblood, Anna M. Schulz, Alfred Zippelius, Percy A. Knolle, Matthias Heinig, Carl-Philipp Hackstein, Dietmar Zehn

**Affiliations:** 1 Division of Animal Physiology and Immunology, School of Life Sciences Weihenstephan, Technical University of Munich, Freising, Germany; 2 Institute of Computational Biology, Helmholtz Zentrum München Deutsches Forschungszentrum für Gesundheit und Umwelt, Neuherberg, Germany; 3 Department of Informatics, Technical University of Munich, Garching, Germany; 4 Center for Infection Prevention (ZIP), School of Life Sciences Weihenstephan, Technical University of Munich, Freising, Germany; 5 Institute of Molecular Immunology, School of Medicine and Health, Technical University of Munich, Munich, Germany; 6 Department of Immunology, St Jude Children’s Research Hospital, Memphis, TN USA; 7 Cancer Immunology, Department of Biomedicine, University Hospital Basel, Basel, Switzerland; 8 Department of Computer Science, TUM School of Computation, Information and Technology, Technical University of Munich, Garching, Germany; 9 Present Address: Cancer Immunology, Department of Biomedicine, University Hospital Basel, Basel, Switzerland

**Keywords:** Infection, Viral infection

## Abstract

T cell exhaustion limits effector T cell function in chronic infection and tumours^1,2^. The development of these hypofunctional T cells and of their precursors was considered to require stimulatory conditions that are met only after persistent exposure to antigen and inflammation. Here we show, however, that similar T cell populations exist in the early phase of acute infections^1,2^. At that stage, the early developing TCF1^+^ precursor population exhibits an unexpected diversity; it includes precursors of normal memory T cells, but also cells with phenotypic, gene-expression and epigenetic profiles that resemble those of precursors of exhausted T cells found in chronic infections. We show that high ligand affinity promotes and PD-1 signalling restricts the development of these precursors. Although the exhausted precursors are at first found frequently, they decline without being completely lost in infections that the immune system resolves. We therefore conclude that precursor T cells with at least two distinct phenotypes are pre-emptively generated irrespective of the outcome of an infection.

## Abstract (web summary)

T cell exhaustion was thought to be strictly associated only with chronic infections and tumours, but it turns out that acute infections also generate a subset of precursor T cells with exhaustion-like phenotypes.

## Main

T cell exhaustion refers to the formation of hypofunctional T cells with a reduced effector function and reduced potency in eliminating target cells in the setting of prolonged antigen exposure in chronic infections and tumours. Characterized by a reduced cytokine-secretion capacity and upregulated expression of inhibitory receptors (PD-1, LAG3, 4-1BB or OX40)^1–4^, exhausted CD8 T cells also express a unique pattern of transcription factors, which includes high levels of TOX, NR4A1, NR4A2 and EOMES, and lower levels of T-bet (refs. ^5–12^) and of hypoxia-inducible factor 1α (HIF-1α)^13^. Moreover, the hypofunctional phenotype is epigenetically enforced and ‘exhaustion’ imprints are retained even when cells are transferred from chronic into acute infections^14^, and after their reactivation through checkpoint inhibitors^2,15–18^. Although this exhausted state is crucial for preventing immunopathology in the context of chronic antigen stimulation, the low effector capacity of exhausted T cells is a major obstacle for effective immunotherapy against chronic infections and tumours^19–21^. Understanding the kinetics and mechanisms that drive the induction and maintenance of exhausted T cells is therefore essential for devising advanced immunotherapeutic solutions that aim to prevent or overcome their development.

The vast majority of exhausted T cells are terminally differentiated effector cells, which lack the capacity to undergo further expansion. Nonetheless, the long-term maintenance of exhausted T cell populations, which can persist for years in people with tumours or chronic^1,22,23^ hepatitis C infections, requires a population of precursor cells, with a stem-cell-like function. These cells have been shown to be produced in the early phase of chronic infections^24^, but so far, they have been considered to be formed exclusively in chronic infections and tumours, or in infections that the immune system struggles to eliminate—such as severe COVID-19 infections^25^. In sharp contrast to this view, here, by systematically studying early differentiation events in acute infection, we observed substantial heterogeneity in the cell population expressing TCF1, which is thought to give rise to memory precursor cells. We discovered that this population contains a subset that exhibits key features of precursors of exhausted T (T_pex_) cells^1^. These precursors express TOX and PD-1, and have epigenetic and transcriptional profiles similar to those of exhausted T cells. We further show that these T cells are driven by strong TCR stimulation and that their development is promoted by ligand affinity and antagonized by PD-1 signalling. Accordingly, our observations challenge the concept that precursors of exhausted T cells are exclusively generated in chronic infections or tumours. Instead, we show that these cells are pre-emptively or constitutively formed in infections that the immune system successfully eliminates.

## Exhausted T cells exist in acute infections

**Fig. 1:**
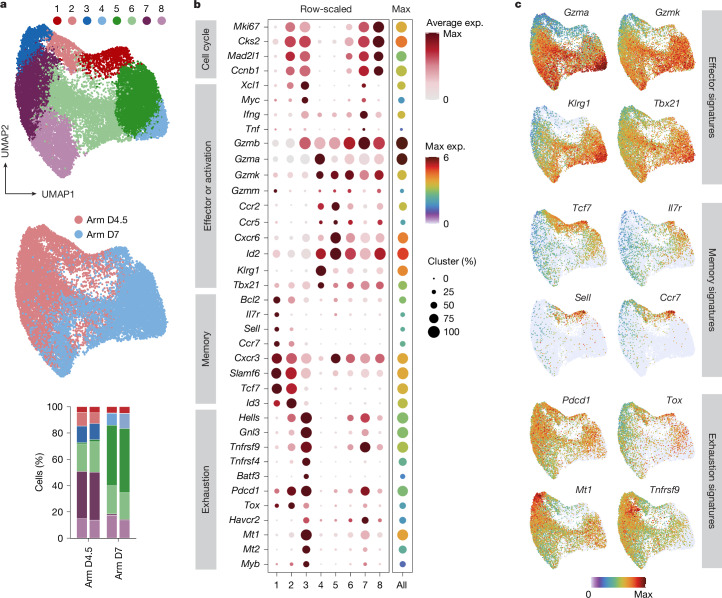
Acute LCMV-specific T cell populations contain cells with transcriptional similarity to exhausted T cells. **a**–**c**, A published dataset^26^ was used in which LCMV-specific, TCR-transgenic P14 T cells were transferred into host mice that were subsequently challenged with acute LCMV Armstrong (Arm) infection. P14 cells were re-isolated on days (D) 4.5 and 7 after infection. **a**, Uniform manifold approximation and projection (UMAP) shows eight identified clusters (top), the distribution of the two time points projected over the Louvain clusters (middle) and an illustration of the relative size of the cluster in each sample (bar graph at the bottom). **b**, Signature dot plot for the clusters. Colours encode average normalized expression values within the clusters, and circle size indicates the percentage of the cluster expressing the gene of interest. **c**, Feature plots illustrating selected effector, memory and exhaustion markers. Colours encode normalized expression values. Data are derived from a single previously published dataset.

Precursors of exhausted effector T cells have been shown to be detectable in infections that become chronic as early as five days after infection^1,24^ and thus long before the infection turns into a chronic phase. This early commitment raised our interest in investigating whether similar cells might also arise in the early phase of infections that eventually resolve. To interrogate cell heterogeneity in an unbiased manner, we used the public single-cell RNA sequencing (scRNA-seq) dataset GSE119943 (ref. ^26^). These data stem from experiments in which lymphocytic choriomeningitis virus (LCMV)-specific, TCR-transgenic P14 T cells were transferred into host mice, followed by an acute LCMV infection. Single-cell-resolved levels of gene expression were determined after isolating the P14 T cells from the spleen on days 4.5 or 7 after infection. By reanalysing the datasets, we identified eight clusters with distinct gene-expression signatures (Fig. 1a,b). Clusters 4–8 seemed to be effector cells typically associated with acute infection, expressing high levels of *Id2* and varying levels of *Tbx21* and effector molecules (*Gzmb*, *Gzma*, *Gzmk*, *Ifng* and *Tnf*). Cluster 4 predominantly included day-7 T cells expressing a transcriptional signature similar to that of terminal effector T cells, with enhanced levels of genes such as *Klrg1* and those encoding granzymes (*Gzma* and *Gzmk*; Fig. 1b,c). Clusters 5 (day 7) and 6 (days 4.5 and 7) showed an early effector phenotype, with expression of granzymes but with lower expression of *Klrg1* compared with the terminal effectors seen on day 7. Clusters 7 (day 4.5) and 8 (days 4.5 and 7) contained high levels of cycling effector cells, with upregulated cell-cycle signatures such as *Mki67*, *Cks2*, *Mad2l1* and *Ccnb1* (Fig. 1b,c), and cluster 7 also expressed the highest levels of *Ifng*, *Tnfa* and *Havcr2*. By contrast, cluster 1, which contained T cells from both day-4.5 and day-7 datasets (Fig. 1b,c) exhibited a transcriptional signature known for memory precursor cells (T_mpc_), which included expression of *Tcf7*, *Il7r*, *Sell* and *Id3* and downregulation of effector molecules such as *Klrg1*, *Gzma*, *Gzmk* and *Tbx21* (Fig. 1b,c). Cluster 2 (day 4.5) contained a combination of markers of progenitor cells (*Tcf7*, *Slamf6* and *Id3*) and markers of T cell exhaustion (*Xcl1*, *Pdcd1* and *Tox*). T cells in the day-4.5 restricted cluster 3 (Fig. 1b) expressed high levels of early activation markers, including *Xcl1* and *Myc*, high levels of well-known genes associated with exhaustion, such as *Pdcd1*, *Tox*, *Tnfrsf9* (4-1BB), *Myb* and *Havcr2* (TIM3), low levels of traditional effector molecules (*Id2*, *Tbx21*, *Cxcr6* and *Ccr5*) and reduced levels of granzymes (*Gzma*, *Gzmk* and *Gzmm*) (Fig. 1b,c). Thus, the phenotype of cells found in clusters 2 and 3 suggests that cells with transcriptional similarity to that seen in T cell exhaustion are detectable in the early phase of acute infection. This encouraged us to examine the origin and fate of these cells in more detail.

## T_ex_ precursors in acute infections

**Fig. 2:**
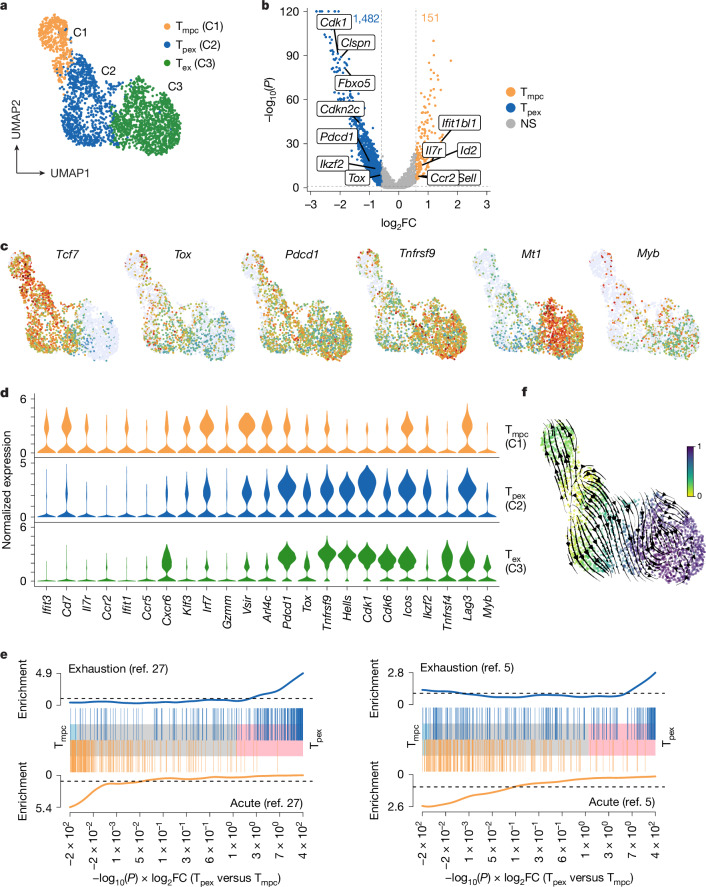
Cells similar to memory precursor T cells and to exhausted precursors exist early in acute infection. Day-4.5 cells from Fig. 1 located in progenitor clusters 1 and 2 and T_ex_ cluster 3 were selected, re-clustered and analysed. **a**, T_pex_, T_mpc_ and T_ex_ clusters after re-clustering. **b**, Volcano plot of differential gene expression between the T_mpc_ cluster and the T_pex_ cluster (as shown in **a**). Genes with *P*_adj_ < 0.05 and log_2_-transformed fold change (log_2_FC) > log_2_(1.5) were considered differentially expressed. Significance was calculated with Wilcoxon rank-sum test and adjusted for multiple testing with Benjamini–Hochberg correction. **c**, Feature plots illustrating the representative markers. Colours encode normalized expression values. **d**, Violin plots showing expression levels of selected signature genes by cells assigned to the new clusters depicted in **a**. **e**, GSEA for exhausted (top) and acute (bottom) signatures from two distinct sources (left, ref. ^27^; right, ref. ^5^) on T_pex_ (cluster 2) versus T_mpc_ (cluster 1). The background genes were sorted by differential expression levels between these two clusters, weighted by the significance obtained with a Wilcoxon rank-sum test. **f**, Combined velocity and pseudotime analysis using the subclusters shown in **a**. Data are derived from a single previously published dataset.

**Extended Data Fig. 1:**
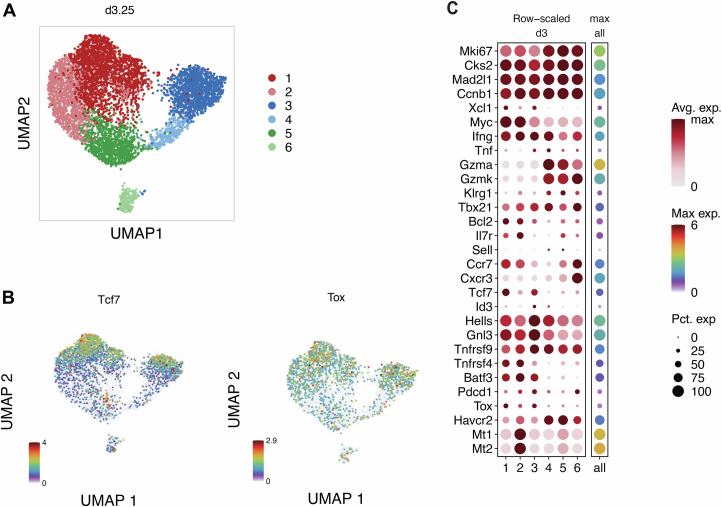
Precursor populations present at 3.25 days after infection show only minimal differences in the expression of T_pex_-associated genes. **a**, TCR-transgenic P14 T cells were re-isolated from LCMV ARM infected host mice at day 3.25 after infection and subjected to scRNA-seq. Depicted are the six Louvain clusters identified at this time point. **b**, Feature plots showing the expression of *Tcf7* and *Tox* in the clusters as in **a**. **c**, Average cluster expression of genes associated with CD8 T cell differentiation, effector function and memory. Data were derived from a single experiment.

To investigate the clusters showing signs of exhaustion in more detail, we specifically reanalysed the *Tcf7*-overexpressing precursor clusters 1 and 2 and the exhausted cluster 3. (Fig. 2a). Differential expression analysis comparing the two precursor clusters identified 1,482 genes that were upregulated in cluster 2 compared with cluster 1. Because *Tox*, *Lag3*, *Pdcd1*, *Ikzf2*, *Tnfrsf9* and *Myb* were detected in cluster 2, we termed these cells precursors of exhausted T (T_pex_) cells (Fig. 2a–c). By contrast, among the 151 upregulated genes in cluster 1, we identified some that are typically associated with conventional memory cells. We therefore refer to these cells as memory precursor T (T_mpc_) cells (Fig. 2b). Of note, T_pex_ cells shared several features with the day 4.5 exhausted T (T_ex_) cells in cluster 3 that we had included in our analysis as a reference population (Fig. 2c,d). To further examine differentially expressed signatures identified in two types of precursors, we extracted acute and exhausted signatures generated from two previously published studies^5,27^. Gene set enrichment analysis (GSEA) revealed that the cells from cluster 2 showed an enrichment of exhaustion gene signatures, whereas those in cluster 1 showed an enrichment of the corresponding gene signatures derived from cells from an acute infection (Fig. 2e). We also performed a pseudotime and velocity analysis on the selected clusters (Fig. 2f). The lowest pseudotime values were observed at the border between the T_pex_ and the T_mpc_ clusters and increased from there into the far ends of both clusters, suggesting that unique differentiation avenues result in the existence of these two types of precursor cells. Pseudotime further increased from the T_pex_ to the T_ex_ cluster, and plateaued in the latter, positioning the T_ex_ population downstream of the T_pex_ one. Because these results pointed towards the existence of a common precursor of T_mpc_ and T_pex_, we screened for such a cell before day 4.5. ScRNA-seq of P14 T cells that were re-isolated at day 3.25 after infection revealed two different *Tcf7*-expressing precursor clusters (Extended Data Fig. 1a,b). Notably, these populations showed only marginal differences in the expression of genes such as *Pdcd1*, *Tnfrsf9*, *Ccr7* and *Il7r*, and no differences in the expression of *Tox* (Extended Data Fig. 1b,c). This shows that distinguishable T_pex_ and T_mpc_ populations arise after day 3.25 and are visible at day 4.5 after infection. Altogether, our analysis reveals that the early precursor population contains not only cells that are related to classical memory precursor cells, but also a subpopulation that resembles the T_pex_ cells found in chronic infections.

## Exhaustion markers in early T_pex_ cells

**Fig. 3:**
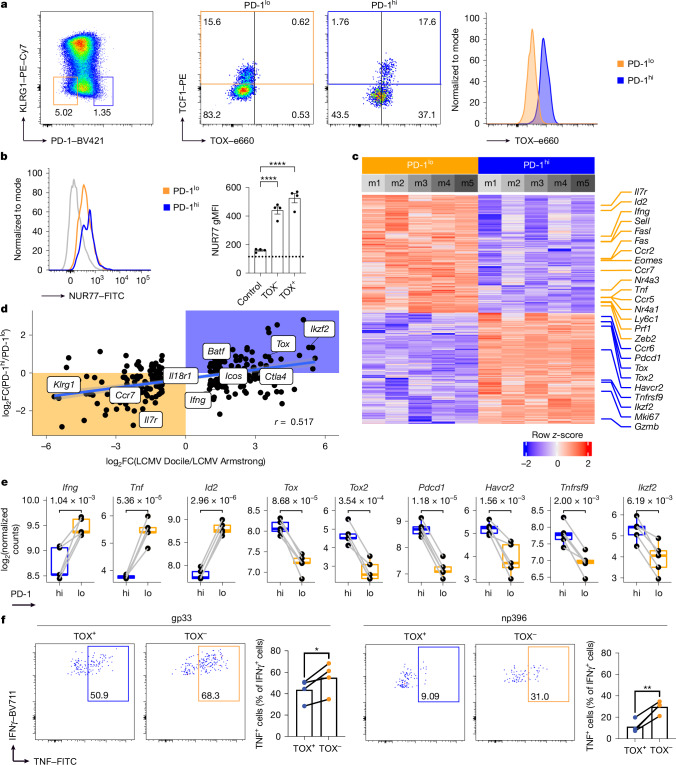
Transcriptional changes between PD-1^hi^ and PD-1^lo^ precursors reveal an exhausted phenotype. **a**, CD45.1 congenic, TCR-transgenic P14 (10^3^) T cells were transferred into C57BL/6 hosts, which were then infected with LCMV Armstrong. CD44^+^KLRG1^−^PD-1^hi^ and PD-1^lo^ populations were selected and analysed for TCF1 and TOX expression seven days after infection. **b**, NUR77 expression in PD-1^hi^ and PD-1^lo^ subsets of transferred P14 T cells on day 4.5 after infection with LCMV Armstrong. *n* = 4; mean ± s.e.m.; ****P < 0.0001 (ordinary one-way ANOVA with Dunnett’s multiple comparisons test). gMFI, geometric mean fluorescence intensity. **c**, Heat map of differentially expressed genes (*P*_adj_ < 0.05 and |log_2_FC| > 0.58) between PD-1^hi^ and PD-1^lo^ P14. **d**, Correlation of the log_2_FC between PD-1^hi^ and PD-1^lo^ P14 with the log_2_FC between acute LCMV Armstrong and chronic LCMV Docile infection at day 21 after infection. Line represents a linear model regression and the bands show a confidence interval of 95%. **e**, Log_2_-normalized counts of bulk RNA-seq with paired PD-1^hi^ and PD-1^lo^ P14 samples from the same donor mouse. Significance values were calculated using empirical Bayes moderated *t*-statistics and were adjusted with Benjamini–Hochberg correction. *n* = 5 for all groups; lines represent mean and symbols represent individual mice. Box plots show median, first and third quartiles (hinges); lines show the smallest or largest observation within a distance from the nearest hinge of 1.5 times the size of the box. Observations outside this range are shown as outliers. **f**, Comparison of TNF co-production by INFγ^+^ cells after brief ex vivo stimulation with gp33 or np396 peptide. Spleen cells were isolated on day 4.5 after infection with LCMV Armstrong and pre-gated as KLRG1^−^TCF1^+^TOX^+^ (TOX^+^) or KLRG1^−^TCF1^+^TOX^−^ (TOX^−^) T cells. *n* = 4; *P = 0.0417, **P = 0.0046 (two-tailed paired *t*-tests). Data are representative of three (**a**) or two (**b**,**f**) independent experiments or derived from a single experiment (**c**–**e**).

**Extended Data Fig. 2:**
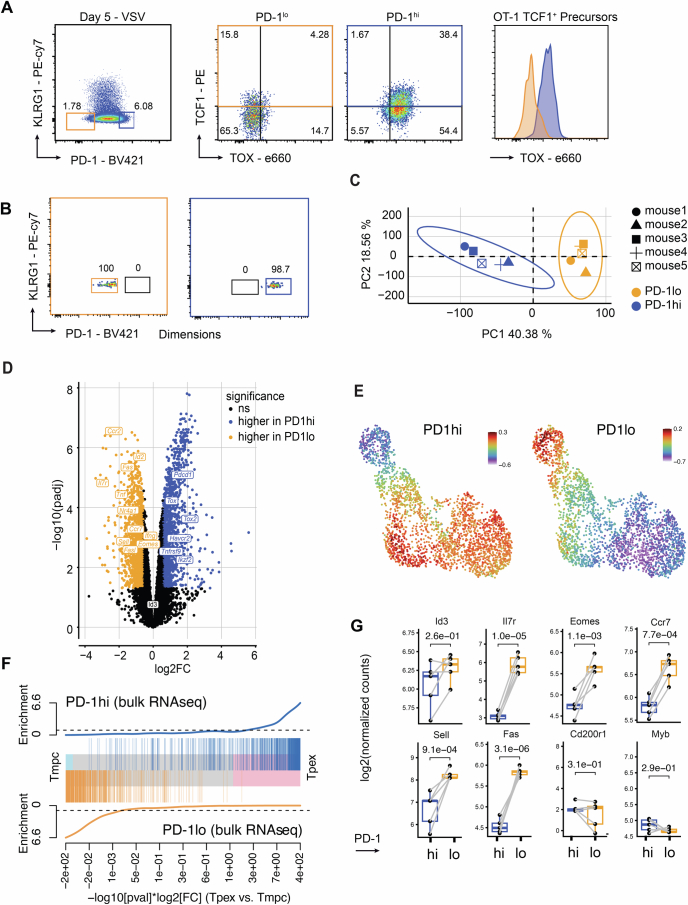
Transcriptional changes between PD-1^hi^ and PD-1^lo^ precursors are strong and match differences seen in comparisons of LCMV-specific CD8 T cells isolated from chronic versus acute infections. CD45.1 congenic OT-1 (5 × 10^3^) T cells were transferred into C57BL/6 hosts, which were then infected with recombinant ovalbumin expressing vesicular stomatitis virus (VSV). **a**, Expression of TCF1 and TOX were analysed in PD-1^hi^ (blue) or PD-1^lo^ (yellow) KLRG1- OT-I cells from VSV-infected mice. **b**, Adoptively transferred Tcf7^gfp(bright)mCherry^ reporter transgenic P14 T cells were re-isolated from LCMV-infected mice on day 7 after infection and separated into GFP^+^ CD44^+^ KLRG1^−^ PD-1^lo^ vs PD-1^hi^ cells and submitted for sequencing. **c**, Samples of PD-1^hi^ and PD-1^lo^ precursors segregate along principal component 1. **d**, Volcano plot comparing PD-1^hi^ and PD-1^lo^ samples. Significance values were calculated using empirical Bayes moderated t-statistics and were adjusted with Benjamini–Hochberg correction. **e**, Signature score of genes associated either with PD-1^hi^ or PD-1^lo^ CD8 T cells projected onto the UMAP from Fig. 2a. **f**, Gene set enrichment analysis of signatures from PD-1^hi^ and PD-1^lo^ samples on T_pex_ (cluster 2) vs. T_mpc_ (cluster 1) from Fig. 2a. The background genes were sorted by differential expression levels between these two clusters, weighted by their significance. *P* values were calculated using Wilcoxon rank-sum test. **g**, Log_2_ normalized counts of bulk RNA-seq with paired PD-1^hi^ and PD-1^lo^ P14 samples from the same donor mouse. Significance values were calculated as in **d**. n = 5 for all groups, lines in **g** represent the mean and symbols identify individual mice. Box plots show median, first and third quartiles (hinges), the lines show the smallest or largest observation within a distance from the nearest hinge of 1.5 times the size of the box. Observations outside this range are shown separately as outliers. Data were derived from three independent experiments (**a,b**) or a single experiment (**c-g**).

**Extended Data Fig. 3:**
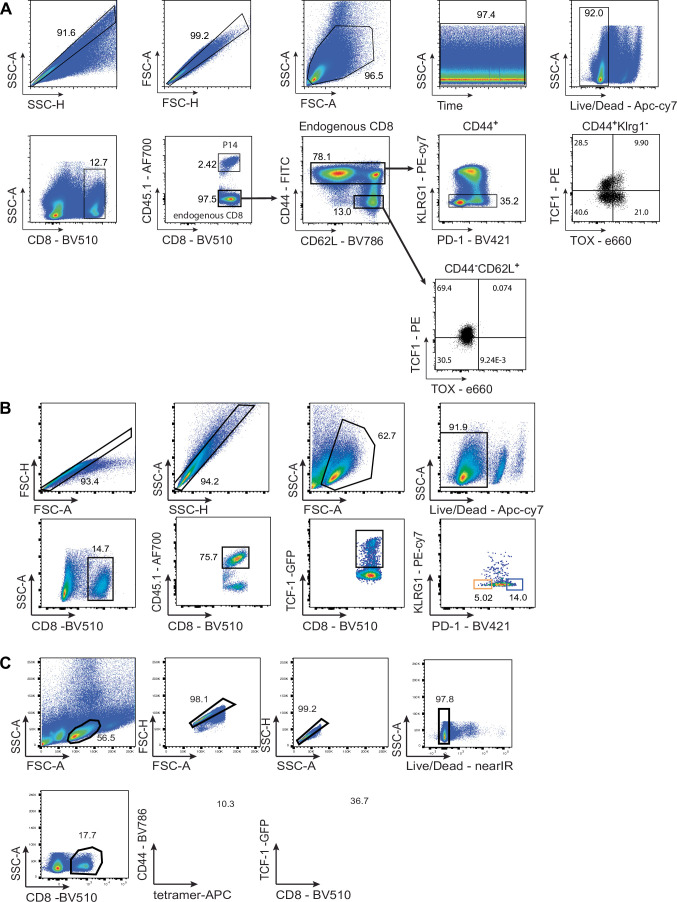
Gating strategies for FACS analyses and cell sorting. **a**, Gating strategy to analyse expression of TCF1 and TOX (Fig. 3a,b, Fig. 5a–d and Extended Data Fig. 2a) or the detection of IFNγ and TNF within the TOX+ and TOX- TCF1+ cells (Fig. 3f). Cells were gated for singlets/fsc-ssc/time/live/CD8^+^/CD45.1^−^ or CD45.1^+^/CD44^+^/KLRG^−^ followed by analysis of TCF1 vs TOX. TCF1 and TOX gates were also applied on the endogenous CD44^−^CD62L^+^ naive population to confirm proper placement of TOX gates. **b**, Gating strategy for the sort of precursor populations (Fig. 3c–e and Extended Data Fig. 2b–d,g,h) Cells were purified by MACS column (Miltenyi) through positive selection of CD45.1^+^ cells and then stained and sorted for singlets/fsc vs ssc/live/CD8^+^/CD45.1^+^/TCF1_GFP^+^ and for PD-1^hi^ (blue) vs PD-1^lo^ (orange). For the sort of total P14 cells (Extended Data Fig. 1) the same strategy was used. Here, within the singlets/fsc-ssc/live/CD8^+^ population the CD45.1^+^ cells were sorted. **c**, Gating strategy for the sort of endogenous precursor populations (Fig. 7c–e and Extended Data Fig. 6). Cells were gated for fsc-ssc/singlets/live/CD8^+^/CD44^+^ tetramer^+^/TCF1-GFP^+^. The same gating strategy without the last gate was also used for the analysis of TOX and TCF1 in endogenous tetramer^+^ populations (Fig. 7a,b).

Next, we sought to confirm the presence of T_pex_ cells through flow-cytometry-based analysis in various acute infections. We transferred P14 or OT-1 TCR-transgenic T cells into CD45.1 congenic host mice, which were subsequently infected either with the acute LCMV Armstrong strain (Fig. 3a) or with a recombinant vesicular stomatitis virus that expresses ovalbumin (VSV-N4Ova) (Extended Data Fig. 2a). Transgenic T cells were re-isolated either five or seven days after infection and analysed by flow cytometry. We excluded KLRG1-positive terminally differentiated cells and selected from the remaining cells the population with the highest or lowest PD-1 expression (Fig. 3a and Extended Data Fig. 2a). To identify TCF1- and TOX-positive cells, we established a gating strategy (shown in Extended Data Fig. 3). We used total endogenous CD8 T cells as reference. We gated on CD44^hi^ cells, and selected KLRG1^−^ cells. The resulting cells revealed clear TCF1 populations that showed robust differences in TOX expression. Moreover, endogenous CD44^−^CD62L^+^ naive cells acted as an internal negative control for TOX expression (Extended Data Fig. 3). Applying these gates to the P14 and OT-1 T cells (Fig. 3a and Extended Data Fig. 2a), we found that PD-1^hi^ and PD-1^lo^ cells differed significantly in their expression of TOX, which was high in the PD-1^hi^ group (Fig. 3a and Extended Data Fig. 2a). Moreover, both PD-1^hi^ and PD-1^lo^ cells contained a notable population of TCF1^+^ precursor cells expressing TOX in the PD-1^hi^ but not in the PD-1^lo^ group (Fig. 3a and Extended Data Fig. 2a). This indicates that cells expressing signs of exhaustion and markers of progenitor cells can also be found using flow-cytometry analysis. Of note, upregulation of NUR77 in both PD-1^hi^ and PD-1^lo^ P14 T cells suggests that both subsets were activated and received recent TCR signalling (Fig. 3b). NUR77 levels were slightly higher in the PD-1^hi^ P14 T cells, which indicates that the PD-1^hi^ phenotype is specifically induced in T cells receiving higher levels of TCR signalling.

To further examine the diversity between the two types of precursors, we used a previously generated P14 Tcf7^gfp(bright)mCherry^ reporter mouse that shows very bright GFP fluorescence, compared with a previously generated mouse^17^, and enables a better selection of early TCF1-expressing precursors. For technical reasons, the P14 TCF1 reporter cells were adoptively transferred into Vβ5 TCRβ-chain-only transgenic hosts to prevent the rejection of transferred mCherry-expressing reporter cells during LCMV infections. These mice contain a polyclonal but restricted TCR repertoire. They have endogenously rearranged TCRα chains but they express the TCRβ chain that is also contained in OT-1 TCR-transgenic mice. This fixed TCRβ chain biases the TCR repertoire and prevents rejection of the Tcf7^gfp(bright)mCherry^ cells. Host mice were then infected with LCMV seven days after infection. TCF1-expressing P14 precursors were isolated, sorted into PD-1^lo^ or PD-1^hi^ precursors (Extended Data Fig. 2b) and submitted for RNA-seq. Principal component analysis (PCA) showed that the component capturing most of the variance in the data corresponds to the PD-1 status of the samples (Extended Data Fig. 2c). We performed differential gene-expression analysis comparing the PD-1^hi^ with the PD-1^lo^ precursors and found a total of 2,550 differentially expressed genes. Of these, 1,236 genes were more highly expressed in the PD-1^hi^ population and 1,314 in the PD-1^lo^ population (*P*_adj_ < 0.05 and log_2_FC > log_2_(1.5)) (Fig. 3c and Extended Data Fig. 2d). Next, we used a different public Gene Expression Omnibus (GEO) dataset (GSE142687) to compare the transcriptional differences between our PD-1^hi^ and PD-1^lo^ precursors with the differences between precursors taken from chronically infected LCMV Docile and acutely infected LCMV Armstrong at day 21 after infection^24^. Transcriptional changes of the two comparisons correlated with *r* = 0.517 (Fig. 3d). GSEA using lists of genes significantly upregulated in PD-1^hi^ or PD-1^lo^ cells showed an enrichment in T_pex_ or T_mpc_, respectively (Extended Data Fig. 2e,f). All of these observations show that PD-1^hi^ sorted precursors share a strong exhaustion signature, as seen in the T_pex_ cells described in chronic infections^1,24^. In addition, we found that PD-1^hi^ precursors transcribed increased levels of receptors associated with exhaustion (*Pdcd1*, *Havcr2* and *Tnfrsf9*) as well as other genes that are significantly upregulated in exhaustion, such as *Tox*, *Tox2* and *Ikzf2* (Fig. 3e). By contrast, PD-1^lo^ precursors shared characteristics with conventional memory cells, with high expression of *Il7r*, *Eomes*, *Ccr*7 and *Id3* (Extended Data Fig. 2g). Among the genes that are differentially expressed between the two types of precursors, we noted that PD-1^hi^ precursors transcribed lower levels of *Id2* and of effector cytokines (*Ifng* and *Tnf*) (Fig. 3e). To confirm the reduced expression of effector cytokines at the protein level, we isolated total splenocytes on day 4.5 after infection and briefly re-stimulated ex vivo using LCMV-derived gp33 and np396 peptides. Again, we found that T_pex_-like cells showed an impaired capacity to co-produce TNF and IFNγ on in vitro re-stimulation, compared with their T_mpc_ counterparts (Fig. 3f). Together, the presented data led us to conclude that precursor cells resembling chronic-infection-associated T_pex_ cells are formed in the early phase of acute infections. Furthermore, the data show that a diverse set of precursor T cells is generated in the early phase of infections independently of whether or not the infection eventually becomes chronic or resolved.

## Epigenetics of T_pex_ cells in acute infections

**Fig. 4:**
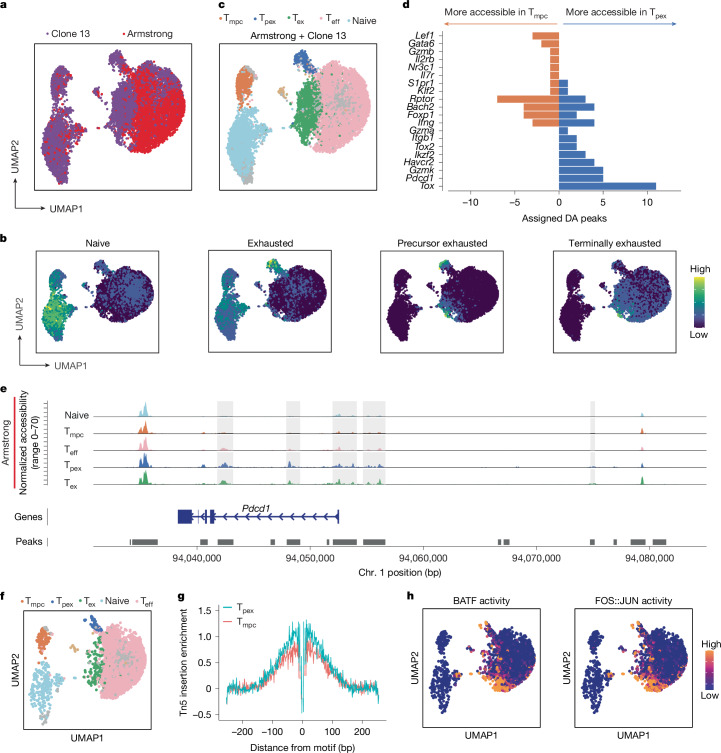
T_pex_ cells from acute infections epigenetically resemble their counterparts from chronic infections. Public scATAC-seq data^28^ for total splenic CD8 T cells analysed seven days after mice were infected with either LCMV Armstrong or LCMV clone 13. **a**, UMAP embedding of cell similarity. Cells are coloured by infection type. **b**, Signatures for naive, exhausted, precursor exhausted (T_ex_) and terminally exhausted cells derived from bulk ATAC-seq data are used to compute signature scores for each cell. Colours represent the mean expression level of the signature within the cell shown on the UMAP embedding. **c**, Cell-type annotations were added on the basis of reference signatures derived from the bulk ATAC-seq experiments in **b**. T_eff_, effector T cells. **d**, Number of differentially accessible (DA) regions between T_pex_ and T_mpc_ annotated to genes of interest. **e**, *Pdcd1* locus accessibility signal of cells from acute infection. Marker regions of the T_pex_ cluster are highlighted in grey. **f**, UMAP embedding of cell similarity subset on cells from Armstrong infection only. Colours represent cell assignments to Leiden clusters. **g**, Accessibility signal at FOS::JUN motif locations. **h**, Transcription-factor activity for the FOS::JUN motif. Data are derived from a single previously published dataset.

A key element of T_pex_ commitment is the epigenetic imprinting of the exhausted phenotype^14–18^. Accordingly, after identifying T_pex_ cells through flow analysis and transcriptional profiling, we were wondering whether the T_pex_ cells found in early acute infection show the epigenetic landscape of chronic-infection-associated T_pex_ cells. To this end, we used the public dataset GSE164978. In this study, C57BL/6J mice were infected with either LCMV Armstrong or LCMV clone 13. Seven days later, total CD8 T cells were isolated from spleen and analysed by single-cell-resolved assay for transposase-accessible chromatin with sequencing (ATAC-seq)^28^. As a first step in our analysis of the published datasets, we jointly clustered cells from both types of infection (Fig. 4a). In the original publication, the authors had mined numerous ATAC-seq datasets obtained by sequencing bulk populations of sorted exhausted T cells (T_ex_), precursors of exhausted T cells (T_pex_) and terminally exhausted T cells, but also naive cells and memory precursor cells (T_mpc_)^28^.

**Extended Data Fig. 4:**
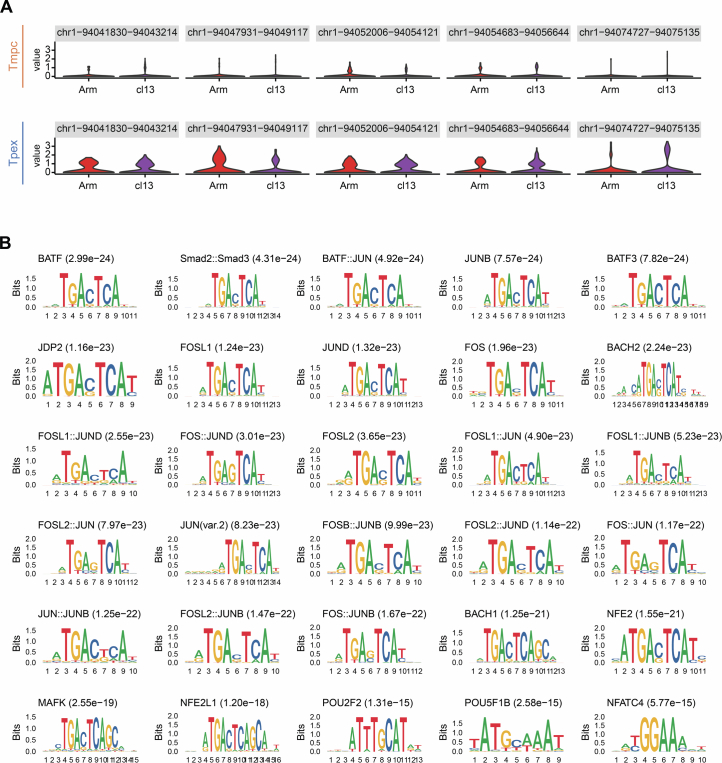
Accessibility at *Pdcd1* locus and motif analysis. **a**, Accessibility levels of T_pex_ marker regions at *Pdcd1* locus (highlighted regions in Fig. 4d) directly comparing T_pex_ and T_mpc_ clusters of Armstrong and clone 13 infections. **b**, Top 30 most differentially active transcription factors comparing T_mpc_ and T_pex_ clusters of Armstrong infection. Data were derived from a single previously published experiment.

We then superimposed these reference bulk signatures onto our clustered single-cell-resolved ATAC-seq analysis (Fig. 4b). We saw a significant overlap between cells showing high signal intensities for the naive and for the T_mpc_ signature. This is in line with the fact that both populations are known to share a large fraction of their epigenetic profiles and transcriptional mechanisms. Similarly, the population that had the strongest T_pex_ signature scored highly in the T_mpc_ signature, again reflecting partially shared epigenetic profiles. On the basis of the results shown in Fig. 4b, we then assigned clusters as naive cells, T_mpc_, T_pex_, T_eff_ and T_ex_ (Fig. 4c). As a final step, we performed differential accessibility analysis between the T_pex_ and T_mpc_ clusters and aggregated differentially accessible regions on the basis of the gene they annotate to (Fig. 4d). As expected, we found that T_pex_ cells are more accessible at several regions in proximity to *Tox*, *Tox2* and *Pdcd1*, compared with T_mpc_ (Fig. 4d). Notably, we observed that the T_pex_ cluster contained cells from both acute and chronic infections (Fig. 4a). Moreover, when directly comparing the accessibility of T_pex_ marker regions in the *Pdcd1* locus of cells isolated from Armstrong or clone 13 infected mice, we did not find significant differences (Extended Data Fig. 4a). This underlines that T_pex_ cells from both acute and chronic infection share similar epigenetic profiles. As a next step, we selected the cells infected with LCMV Armstrong only and plotted the coverage at the *Pdcd1* locus split by cluster assignment (Fig. 4e). The plots highlight in grey the regions identified on the basis of differential accessibility between the T_pex_ and the T_mpc_ cluster. We observed similar accessibility between T_ex_ and T_pex_ and clear differences between T_pex_ and T_mpc_, indicating that differences between T_pex_ and T_mpc_ can be reproduced with cells originating solely from acute infection (Fig. 4f). We continued to use the subset of cells obtained from Armstrong infection and integrated data on known transcription-factor-binding motifs to find putative regulators that drive the differences between T_pex_ and T_mpc_. Among the marker regions associated with higher accessibility in T_pex_ than in T_mpc_, a large portion were part of the AP-1 family of transcription factors (Extended Data Fig. 4b). We see that regions around FOS::JUN sites are more accessible in T_pex_ than in T_mpc_ (Fig. 4g), and infer higher transcription-factor activity (Fig. 4h). Altogether, these observations show that epigenetic marks that are typically associated with T_pex_ cells can also be detected in the T_pex_ population that is found in the early phase of acute infection.

## TCR affinity and PD-1 affect T_pex_ formation

**Fig. 5:**
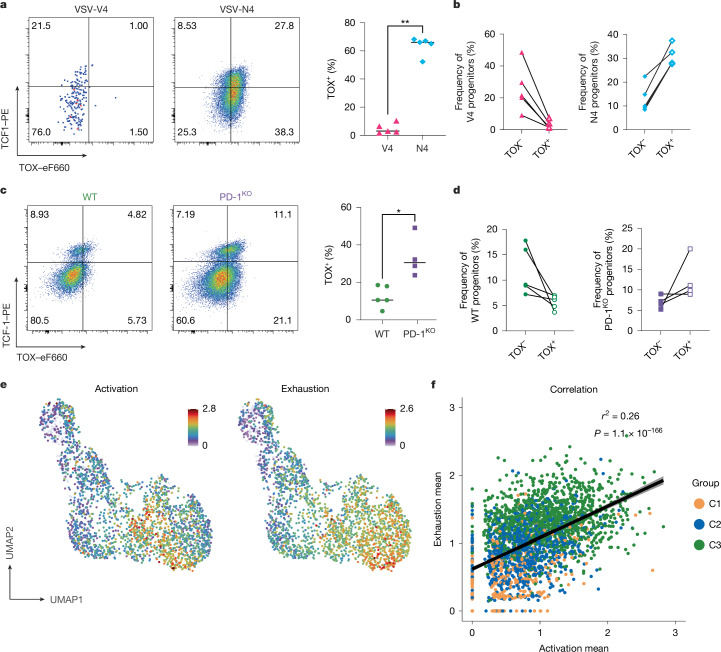
Strong TCR stimulation and the absence of PD-1 signalling promote T_pex_ formation in acute infection. **a**–**d**, Wild-type (WT) OT-1 (**a**,**b**) and wild-type or PD-1^KO^ P14 transgenic (**c**,**d**) T cells were adoptively transferred into CD45.2 congenic hosts. Mice were subsequently infected with VSV-V4 (low-affinity stimulation) or VSV-N4 (high-affinity stimulation) (**a**,**b**) or with LCMV Armstrong (**c**,**d**). Splenocytes were collected seven days after infection. *n* = 5 (OT-I and wild-type P14 recipients); *n* = 4 (PD-1^KO^ recipients). **a**,**c**, Flow plots show TCF1 versus TOX expression by CD44^+^KLRG1^−^ gated OT-1 (**a**) or P14 (**c**) T cells, and graphs show the frequency of TOX^+^ OT-1 (**a**) or P14 (**c**) precursor T cells. *P < 0.05, **P < 0.01 (two-tailed Mann–Whitney test). **b**,**d**, Frequency of TOX^−^ and TOX^+^ TCF1^+^KLRG1^−^ OT-1 (**b**) and P14 (**d**) T cells. **e**, Average normalized expression of genes from activation and exhaustion signatures (Extended Data Table 1) projected onto the UMAP embedding shown in Fig. 2. **f**, Pearson correlation between activation and exhaustion scores for individual cells. Colours encode the subcluster assignment as shown in Fig. 2. Line represents a linear model regression and the bands show a confidence interval of 95%. Each symbol in **a**–**d** represents an individual mouse. Data in **a**–**d** are representative of at least three independent experiments. Lines in **a**,**b** represent the mean. Lines in **b**,**d** mark cells originating from the same donor. Data in **e**,**f** are derived from a single previously published dataset.

Because increased TCR signal strength drives exhaustion in chronic infection^17,29–31^ we sought to determine how ligand affinity and the reception of inhibitory signals affect T_pex_ activation in acute infection. To do so, we used a previously established system in which OT-1 T cells are exposed either to their native ligand SIINFEKL (referred to as N4) or to a lower-affinity altered peptide ligand (SIIVFEKL; V4) during an infection with recombinant vesicular stomatitis virus expressing N4 (VSV-N4) or V4 (VSV-V4)^1^. Mice infected with high-affinity VSV-N4 had a large shift in the frequency of TOX^+^ precursors and effectors at seven days after infection, whereas mice infected with VSV-V4 had little to no TOX^+^ expression in either the precursors or the effector compartment (Fig. 5a,b), indicating that the formation of T_pex_ in acute infections depends on receiving strong TCR signals.

A key feature of T_pex_ cells is the upregulation of PD-1. Being an inhibitory receptor, PD-1 is thought to modulate signals that promote activation through the TCR and CD28 by engaging its ligands^32^. Therefore, we hypothesized that PD-1 expression might limit the extent of T_pex_ formation. To test this, we transferred wild-type or PD-1-knockout (PD-1^KO^) P14 transgenic CD8 (CD45.1) T cells into B6 hosts (CD45.2) and infected these mice with LCMV Armstrong. Samples were analysed by flow cytometry seven days after infection. When comparing PD-1^KO^ P14 to wild-type P14, we noted that PD-1^KO^ cells had a higher frequency of total TOX^+^ cells in both the TCF1^+^ precursor and the TCF1^−^ effector population (Fig. 5c). Moreover, we noted a shift in the precursor population from a dominance of TOX^−^ precursors in wild-type cells towards a higher fraction of TOX^+^ precursors in PD-1^KO^ P14 T cells (Fig. 5d). These data were further corroborated by the fact that the single-cell transcriptomic data for day-4.5 Armstrong-infection-derived T cells (presented in Figs. 1 and 2) also show that highly activated cells exhibit a stronger exhausted signal, which confirms the positive correlation between activation strength and the acquisition of exhaustion signatures (Fig. 5e,f and Extended Data Table 1). We therefore concluded that although strong TCR signalling promotes T_pex_ formation in early acute infection, the expression of PD-1 restricts or limits the generation of T_pex_ cells in acute infection.

## Plasticity of early T_ex_ progenitors

**Fig. 6:**
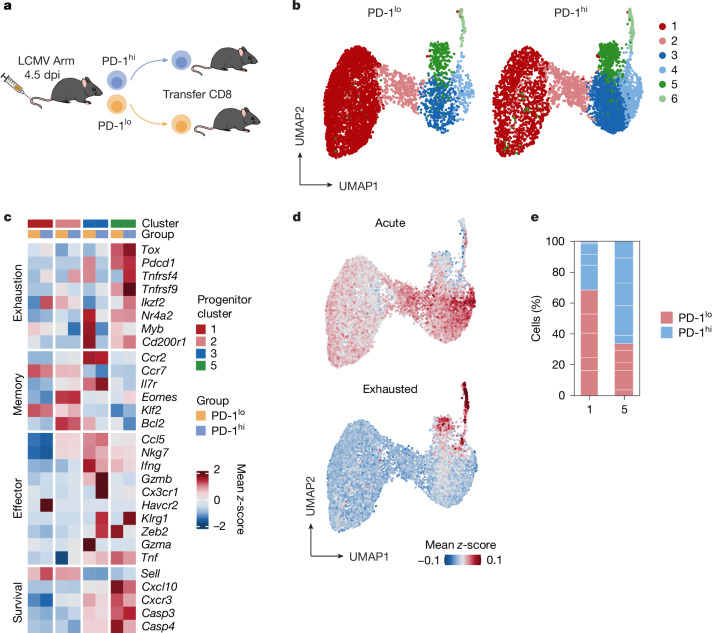
Plasticity of T_pex_ cells formed in the early infection phase. **a**, CD45.1 congenic P14 T cells were transferred into C57BL/6 host mice that were infected with LCMV Armstrong. PD-1^hi^ and PD-1^lo^ P14 cells (gating strategy shown in Extended Data Fig. 3b) were collected at 4.5 days post-infection (dpi) and subsequently retransferred into naive C57BL/6 host mice that were infected with LCMV Armstrong on the same day of P14 transfer. Retransferred P14 cells from PD-1^hi^ versus PD-1^lo^ recipients were collected at 15 days after infection and sent for single-cell sequencing. Graphics were created with Biorender.com. **b**, UMAP representation of Louvain clusters from PD-1^hi^ and PD-1^lo^ recipients. **c**, Heat map showing *z*-scored gene expression segregated by PD-1^hi^ and PD-1^lo^ samples. **d**, Signature score of genes associated with either acute or exhausted CD8 T cells projected onto the UMAP. **e**, Fractions of PD-1^hi^ and PD-1^lo^ recipients in clusters 1 and 5. Data are derived from a single experiment.

**Extended Data Fig. 5:**
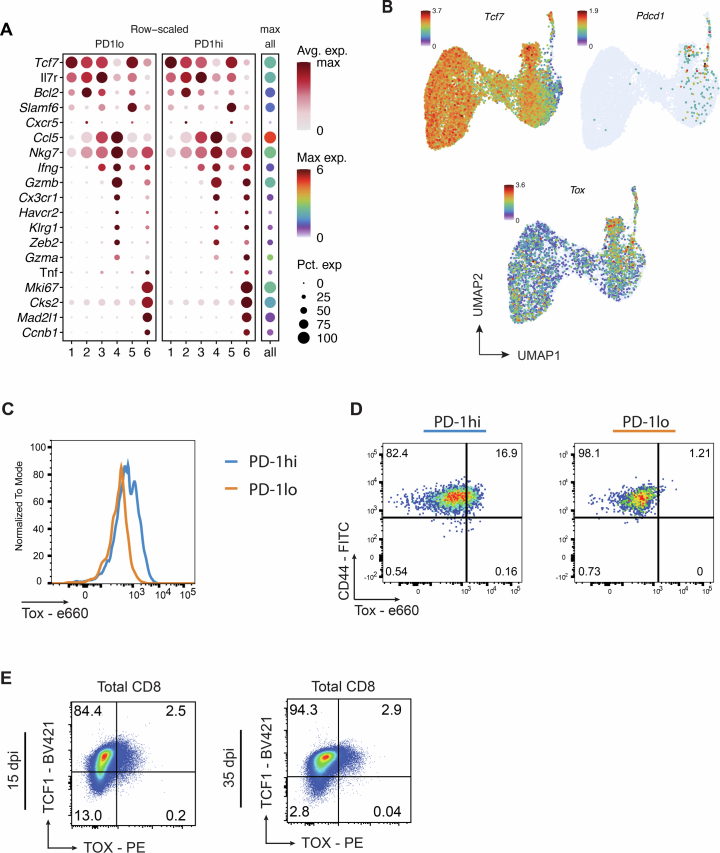
Early but not late T_pex_ cells show developmental plasticity. **a**,**b**, CD45.1 congenic P14 TCR-transgenic T cells were transferred into C57BL/6 host mice as indicated in Fig. 6a. PD-1^hi^ and PD-1^lo^ P14s were re-isolated on day 4.5 after an acute LCMV infection and transferred into a new C57BL/6 host that was then also infected with acute LCMV. 15 days later, the progeny of the PD-1^hi^ and PD-1^lo^ P14 cells were again isolated and analysed by scRNA-seq. **a**, Average cluster expression segregated on PD-1^hi^ and PD-1^lo^. Circle size indicates the percentage of cells expressing the gene of interest. **b**, Feature plots with colours encoding normalized expression values. **c**,**d**, CD45.1 congenic PD-1^hi^ and PD-1^lo^ P14 TCR-transgenic T cells were isolated at 7 days after infection and transferred into naive C57BL/6 hosts. 7 days after infecting the secondary hosts with LCMV Armstrong, the progeny of the transferred PD-1^hi^ and PD-1^lo^ P14 T cells was collected and analysed for TOX and CD44 expression. **c**, TOX expression in PD-1^hi^ and PD-1^lo^ cells. **d**, Co-expression of TOX with CD44 in the indicated cell populations. **e**, C57BL/6 mice were infected with LCMV Armstrong and total splenocytes were isolated 15 and 35 days after infection. Shown are TCF1 and TOX expression by total endogenous CD8 T cells. Data were derived from a single experiment (**a**–**d**) or three independent experiments (**e**).

To determine the stability of T_pex_ cells formed in the early phase of acute infection, we transferred TCF1-reporter-positive P14 PD-1^hi^ or PD-1^lo^ cells (as shown in Fig. 2a) from day-4.5 Armstrong-infected mice into naive hosts. Host mice were then infected with LCMV Armstrong on the same day of transfer (Fig. 6a). scRNA-seq of transferred day-4.5 P14 cells re-isolated at day 15 after infection revealed six distinct clusters (Fig. 6b), spanning the spectrum from cells exhibiting features of progenitors (*Tcf7*, *Il7r* and *Slamf6*; clusters 1, 2, 3 and 5*)* to effector cells that lack progenitor markers and express higher levels of effector function (*Ifng*, *Gzmb*, *Klrg1* and *Cx3cr1*; clusters 4 and 6) (Extended Data Fig. 5a). Of note, the progeny of PD-1^hi^ and PD-1^lo^ cells produced no unique clusters, with both input populations producing cells that were found in all clusters (Fig. 6b). Therefore, to determine whether we could still identify T_pex_, we excluded the effector clusters 4 and 6 and focused our analysis on the progenitor clusters (1, 2, 3 and 5) (Fig. 6c). Progenitor clusters 1 and 2 expressed higher levels of the stemness marker *Sell*, along with increased expression of *Ccr7*. Progenitor cluster 3 expressed higher levels of effector-memory-associated genes, maintaining the highest levels of *Zeb2*, *Gzma*, *Gzmb*, *Cx3cr1* and *Ifng*. Notably, cells found in progenitor cluster 5 were defined by the expression of genes that regulate key features associated with T cell exhaustion and maintenance, including *Tox*, *Pdcd1*, *Ikfz2*, *Tcf1* and *Cd200r1* (Fig. 6c and Extended Data Fig. 5b). To further confirm that the cells in cluster 5 were T_pex_, we analysed the expression of gene signatures associated with either acute or exhausted CD8 T cells; progenitor cluster 5 showed higher expression of exhaustion-associated genes, but lacked genes associated with an acute phenotype (Fig. 6d). Together, the data suggest that early day-4.5 PD-1^hi^ and PD-1^lo^ precursors show considerable plasticity towards their differentiation into various effector and progenitor subsets. Nonetheless, although the progeny of both PD-1^hi^ and PD-1^lo^ P14 cells were found in all clusters (Fig. 6b) and showed comparable gene expression (Fig. 6c), our data also revealed a minor fate bias. We observed, for instance, that in the largest cluster (cluster 1), most cells originated from PD-1^lo^ cells, whereas most cells in T_pex_ cluster 5 were derived from PD-1^hi^ cells (Fig. 6b,e). Furthermore, PD-1^hi^- and PD-1^lo^-derived cells showed some differences in the expression of important exhaustion-associated genes; for example, *Tox*, *Pdcd1* and *Ikzf2* were expressed more highly in PD-1^hi^-derived cells, whereas key effector genes such as *Ifng* and *Tnf* were slightly more highly expressed in the progeny of PD-1^lo^ cells (Fig. 6c). This indicates that features of T cell exhaustion persisted after the transfer. Moreover, we also transferred TCF1-reporter-positive P14 cells with high PD-1 and low PD-1 expression from day-7 Armstrong-infected mice into naive hosts that were then infected with LCMV Armstrong on the day of transfer. Although both types of cells underwent robust secondary expansion, the progeny of the transferred cells still mostly differed in their TOX expression levels (Extended Data Fig. 5c,d). These data show that although there is substantial plasticity at early stages, the T_pex_ phenotype seems to stabilize at later time points.

## T_pex_ stability in acute infections

Next, we sought to determine the longevity and stability of T_pex_ cells. We thought at first that these cells might be only temporarily detectable and that their persistence might require continuous antigen exposure, because we could detect T_pex_ among the sequenced day-4.5 P14 T cells (in Fig. 1) but had difficulties in identifying them in significant numbers among the day-7 cells (Fig. 1a). However, this outcome needs to be viewed with caution as the relatively low sample size of scRNA-seq datasets poses particular challenges in detecting rare subpopulations. Of note, day-7 LCMV-specific T cells contain only around 10% precursor cells, of which only a small fraction might be the descendants of the day-4.5 T_pex_. Thus, the day-7 P14 T_pex_ numbers might be too low to detect them in the scRNA-seq dataset. In fact, rare but detectable numbers of day-7 P14 T_pex_ can be found when large sample sizes and thus larger precursor numbers are analysed by flow cytometry, as shown in Figs. 3a and 5c,d.

**Fig. 7:**
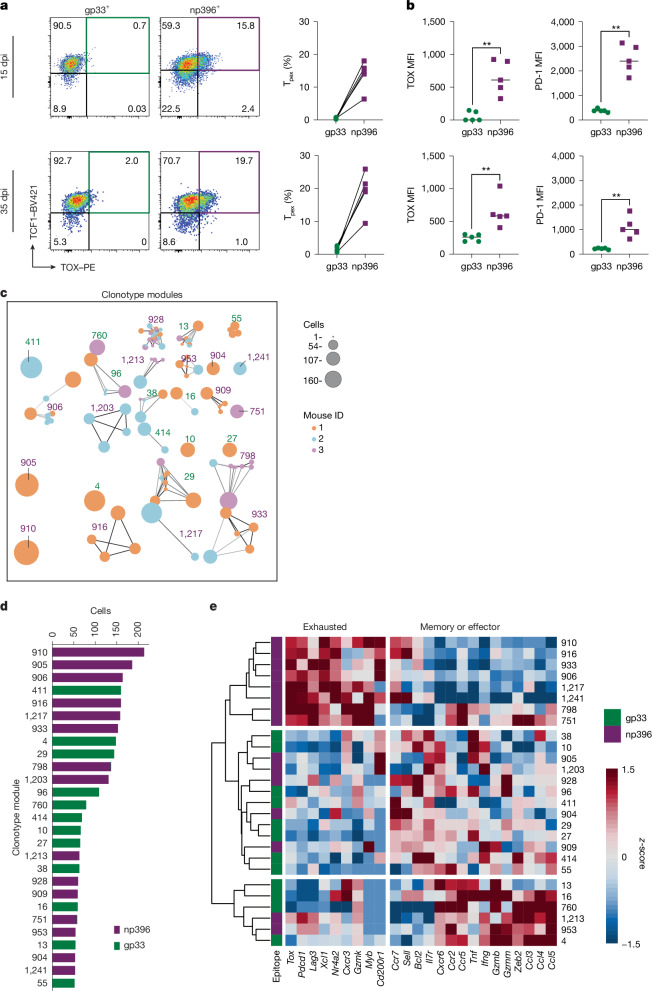
T_pex_ cells are detectable in the memory phase after stronger TCR stimulation. **a**, TCF1 and TOX expression in CD8 T cells stained with gp33^+^ and np396^+^ multimers at 15 or 30 days after infection. Each symbol represents an individual mouse (*n* = 5 mice per group). **b**, Scatter plots showing the mean intensity of expression of TOX (left) or PD-1 (right) by multimer-positive T cells. Each symbol represents an individual mouse (*n* = 5 mice per group); lines represent the mean; *P < 0.05, **P < 0.01 (two-tailed Mann–Whitney test). **c**, gp33-specific (green) and np396-specific (purple) TCF1^+^ precursors were purified from TCF1 reporter mice (*n* = 3) four weeks after LCMV Armstrong infection. Purified cells were subjected to scRNA-seq with paired TCR repertoire analysis. Clonotype pairs were aligned using their amino-acid sequence. Shown are clonotype networks of all modules containing at least 50 cells, with similar clonotypes connected by an edge. Node size depicts clonotype size, the arbitrary clone number, and colours represent mouse ID. **d**, Number of cells per clonotype module. Colours indicate the specificity of the clonotype module. **e**, *Z*-scores of average expression per clonotype module. The dendrogram was generated using Euclidean distance and complete linkage. Data are representative of three independent experiments (**a**,**b**) or derived from a single experiment.

**Extended Data Fig. 6:**
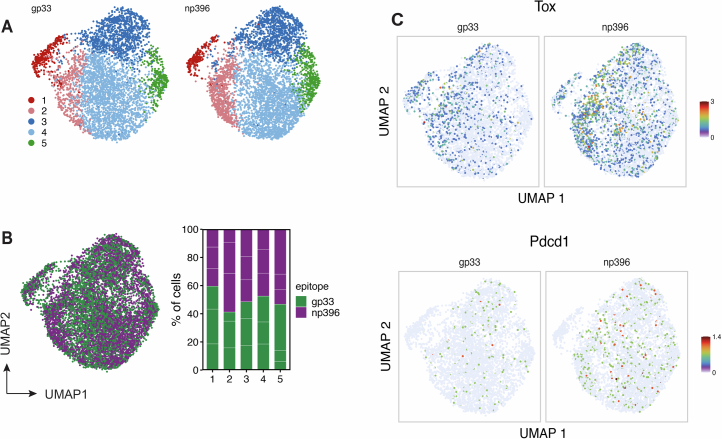
Signs of exhaustion can be found in subsets of np396- but not gp33-specific CD8 T cells. **a**, gp33 and np396-specific TCF1^+^ precursors were purified from TCF1 reporter mice (n = 3) 4 weeks after infecting the mice with LCMV Armstrong. Purified cells were subjected to scRNA-seq. **a**, UMAP representation of Louvain clusters. **b**, Epitope fractions per cluster. **c**, Feature plots depicting the expression of *Tox* or *Pdcd1* in either gp33- or np396-specific CD8 T cells. Data were derived from a single experiment.

Moreover, we noted a considerable number of TCF1- and TOX-co-expressing CD8 T cells of unknown specificity 15 and 35 days after an LCMV infection among endogenous cells (Extended Data Fig. 5e). Cells expressing increased levels of PD-1 and TOX can also be found among CD8^+^ T cells specific to the LCMV-derived np396 epitope at days 15 and 30 after infection (Fig. 7a,b). This indicates that T_pex_ cells that are formed in the early infection period can indeed survive long term and can be found among the memory T cell population. Notably, these cells are detectable only among np396- and not among gp33-multimer-positive populations (Fig. 7a,b). The np396 epitope was previously shown to provide a stronger level of TCR stimulation to T cells than the gp33 peptide^33^. That T_pex_ cells persist among these cells further corroborates our idea that strong TCR stimulation is needed to form and maintain these cells. To characterize this in more depth, we isolated TCF1-expressing memory gp33^+^ or np396^+^ T cells from week-4 LCMV-infected Tcf7^gfp(bright)mCherry^ reporter mice and performed scRNA-seq together with paired α and β TCR repertoire analysis. Clustering identified five populations (Extended Data Fig. 6a), and np396- and gp33-specific cells contributed to all of these in an approximately similar manner (Extended Data Fig. 6b). Although we did not see any clusters that were mostly dominated by either gp33- or np396-specific T cells, we nevertheless noted differences in the expression of key genes associated with T cell exhaustion. In particular, *Tox* and *Pdcd1* were more highly expressed in np396-specific CD8 T cells (Extended Data Fig. 6c). We further noted that *Tox* and *Pdcd1* expression was particularly strong in certain clusters, especially cluster 2, suggesting the existence of a somewhat heterogeneous transcriptional landscape. To analyse this in more depth, we decided to include TCR sequences into our analysis, and aggregated cells into clonotype modules^34^. These are defined as assemblies of TCR nucleotide sequences that are predicted to share similar binding properties to specific antigens and similar TCR signal strength (Fig. 7c). Clonotype modules are grouped on the basis of TCRs that share amino-acid sequences that lead to similar secondary and tertiary structures. Hence, TCR sequences that contain substitutions with functionally equivalent amino acids would be grouped into the same clonotype module, whereas sequences that contain substitutions with amino acids with different biochemical properties would be assigned to another clonotype module. To simplify our findings, we included into our subsequent analysis any module that contained at least 50 cells (Fig. 7c,d). To validate this method, we confirmed that there was no significant sharing of modules between gp33+ and np396+, emphasizing the unique binding properties between gp33+ and np396+ clonotypes (Fig. 7c,d). Of note, we can see module sharing between different mice within certain gp33+ or np396+ clonotypes—confirming that different mice develop unique but also shared TCR amino-acid sequences to the same antigen (Fig. 7c). Aggregating T cells on the basis of clonotype modules showed a clear pattern of modules that express an exhausted signature, including *Tox*, *Pdcd1*, *Lag3*, *Myb* and *Cd200r1* (Fig. 7e). Exhausted modules were specific to np396^+^, whereas the expression of genes associated with effector and acute phenotypes was seen in gp33^+^ and some np396^+^ clonotype modules. (Fig. 7e). Together, these findings are in line with our previous flow cytometric data (Fig. 7a), in which we saw the expression of TOX in some np396^+^ but in very few gp33^+^ CD8 T cells. Taking into consideration our observations that TCR signal strength is crucial for T_pex_ formation (Figs. 3b,  5a,b and 7e) and also that np396 is considered a higher-affinity epitope than gp33 (ref. ^33^), our data strongly suggest that strong TCR stimulation is required for the formation of a long-lived T_pex_ population.

Using a combination of comprehensive sequencing-data analysis and flow-cytometry-based verification, we have shown that T_pex_ cells are generated in acute infections. We show that they are formed in the early phase of infection, and thus long before the infection becomes resolved or chronic. Notably, these T_pex_ cells are found at first in almost as high numbers as typical memory precursor T cells, but they decline substantially thereafter. We take this to mean that the consecutive formation allows the environment to subsequently shape the precursor repertoire on the basis of the infection outcome (Supplementary Fig. 1). Such dynamics have considerable advantages for the infected host, in that they proactively prepare the host for the possibility that the infection becomes chronic, by generating the type of cells that are needed in such infections. This interpretation takes a more recent view of the phenomenon of T cell exhaustion into consideration, which is that forming exhausted T cells can be beneficial for the host^5,20^. Although their reduced effector capacity limits the magnitude by which these cells can protect the host from the pathogen, exhausted T cells are at the same time causing much less pathology than are normal effector T cells^20^. Thus, their presence might ensure that chronic infections are controlled to a necessary level, without causing detrimental organ damage and massive immunopathology.

Mechanistically, we show that T_pex_ formation in acute infections requires strong TCR signals, which are mediated, for instance, by high-affinity TCR ligation. Furthermore, inhibiting T cell activation through PD-1 signalling restricts the formation of T_pex_ cells. The latter is particularly noteworthy, because PD-1 is often thought to induce exhausted T cells. Instead, our data suggest that PD-1 signalling actually prevents exhaustion and limits the formation of T_pex_ cells. Altogether, our observations support a major conceptual change to the commonly held view that exhausted T cells are only formed in the context of long-term antigen persistence. By contrast, we show that the formation of exhausted T cells occurs independently of the outcome of the infection, and that chronic infections propagate and expand these pre-emptively formed T_pex_ cells.

## Methods

### Mice

Mice were bred and housed in specific-pathogen-free facilities at the Technical University of Munich in Germany. P14 TCRαβ-transgenic mice^35^ were provided by A. Oxenius and Vβ5 TCRβ-only transgenic mice^36^ by P. Fink. OT-1 mice were purchased from the Jackson Laboratory. PD-1^KO^ P14 mice were provided by P.-C. Ho. TCF1 reporter mice were generated by us and described previously^19^. Mice were housed under the following conditions: light from 07:00 to 19:00; temperature 22–26 °C; humidity 30–70 g m^−3^. Experiments were performed on male and female mice aged 6–13 weeks in compliance with the institutional and governmental regulations of Germany. They were approved by the responsible veterinarian authorities of the Regierung von Oberbayern in Germany. Statistical methods were not used to predetermine sample size. We chose sample sizes on the basis of previous experience and with the goal of producing statistically robust data while respecting animal welfare regulations. Mice of the appropriate genotype and age were randomly assigned to the experimental groups. We did not perform any readouts involving subjective evaluations, such as histological analysis or clinical scoring. Owing to biosafety regulations, we were instructed not to perform blinding in experiments involving biohazardous substances.

### Viral infections

LCMV virus strains were originally provided by M. J. Bevan. LCMV was expanded in BHK-21 cells and titrated on Vero cells (originally provided by M. J. Bevan) using a focus-forming assay^37^. Recombinant ovalbumin expressing VSV-N4 containing the original SIINFEKL epitope^38^ or the low-affinity altered peptide ligand SIIVFEKL (VSV-V4, originally provided by L. Lefrancois) were expanded and titrated on BHK-21 cells (University of Washington) using a lytic plaque assay^39^. Frozen stocks of LCMV Armstrong, LCMV clone 13 and VSV-N4 or VSV-V4 were diluted in phosphate-buffered saline (PBS). Mice were infected by intraperitoneal injection of 2 × 10^5^ plaque-forming units (PFU) of LCMV Armstrong or intravenous injection of 2 × 10^6^ PFU of recombinant VSV-N4 expressing the SIINFEKL^38^ or VSV-V4 OVA strain displaying the SIIVFEKL epitope. Before experiments, we ensured that the cell lines used showed the expected results and were in good condition. Mycoplasma tests were routinely performed and were negative.

### Cell preparations, T cell purification and adoptive T cell transfers

Single-cell splenocyte suspensions were prepared from naive or infected mice by mashing spleen through a 100-µm cell strainer followed by hypotonic ACK lysis. Total splenocytes were used for flow-cytometry analysis and sorting. P14 TCRαβ transgenic wild-type, P14 TCR-transgenic PD-1^KO^ or OT-1 mice were used as donors for adoptive transfers. CD8^+^ T cells were isolated from splenocyte suspension using the negative selection CD8^+^ T cell enrichment kit II (Miltenyi Biotech) in accordance with the manufacturer’s protocol. For LCMV Armstrong experiments, 1,000 P14 T cells were transferred intravenously by tail-vein injection. For VSV infections, 5,000 OT-1 cells were transferred intravenously by tail-vein injection.

### Flow-cytometry analysis and cell sorting

After isolation, cells were incubated with Zombie NIR dye (BioLegend, 423106) and Fc-blocking reagent 2.4G2 in PBS (Thermo Fisher Scientific) for 15 min at room temperature. Next, cells were washed and resuspended in freshly prepared master mix containing fluorescent staining antibodies in fluorescence-activated cell sorting (FACS) buffer (PBS, 2% fetal calf serum (FCS) and 0.1% sodium azide). When the master mix contained two or more brilliant violet polymer dyes, super bright staining buffer (Invitrogen, SB-4401-42) was used. For tetramer or CXCR5 staining, cells were stained for one hour at room temperature. FoxP3/Transcription factor staining buffer (Thermo Fisher Scientific, 00-5532-00) was used to perform intranuclear staining following manufacturer recommended protocols. Samples were acquired on BD FACS Fortessa or Beckman Coulter CytoFlex LX instruments. For sorting: after live–dead stain and Fc block, cells were incubated with freshly prepared master mix for 20 min at room temperature, followed by washing in magnetic-activated cell sorting (MACS) buffer (PBS, 1% FCS and 2 mM EDTA). Cells were immediately run on a BD FACSAria Fusion sorter. Single-stained controls were prepared using Ultracomp eBeads (Invitrogen 01-2222-42) for every experiment. Beads were treated like the cell-containing samples (including fixation protocol). A detailed list of staining antibodies is provided in Supplementary Table 1. FACS data were analysed using FlowJo software (BD, v.10.09) and exported as Excel (Microsoft, v.16.89.1) tables for subsequent statistical analysis.

### Statistical analysis

GraphPad Prism software was used for statistical analysis (except for the gene-expression data). Student’s *t*-tests (two-tailed) for parametric and Mann–Whitney tests for non-parametric data were used to compare between two independent conditions. Experiments with *n* < 5 were considered non-parametric. When comparing samples originating from the same mouse, a paired Student’s *t*-test was used for parametric data and a Wilcoxon matched pairs signed-rank test for non-parametric data. All measurements were taken from distinct samples.

### De novo 10X Genomics scRNA-seq and data analysis

TCF1 reporter mice were infected directly with LCMV Armstrong. Splenocytes were collected four weeks after infection and stained for np396 and gp33. Tetramer and TCF1-reporter-positive CD8^+^ T cells were purified by flow-cytometry-based sorting. Gene expression and T cell receptor V(D)J libraries were prepared by using the Chromium Next GEM Single Cell 5’ Reagent Kit v2 (PN-1000265, 10X Genomics), Chromium Single Cell Mouse TCR Amplification Kit (PN-1000254, 10X Genomics) and Chromium Next GEM Chip K Single Cell Kit (PN-1000287, 10X Genomics) following the manufacturer’s protocol (CG000331 Rev E). The Dual Index Kit TT Set A (PN-1000215, 10X Genomics) was used for multiplexing (i7 and i5 index read, 10 bp). The samples were sequenced in a paired-end run (read 1: 26 bp; read 2: 90 bp) on a NovaSeq6000 platform using S1 v.1.5 (100 cycles) sequencing kits (20028319, Illumina). Bcl2fastq software (v.2.20.0.422) was used for demultiplexing and generating .fastq files.

### De novo BD Rhapsody scRNA-seq and data analysis

Spleens were collected from LCMV-Armstrong-infected mice at various days after infection (see below) and cut into small pieces. These pieces were then exposed to a medium containing digestive enzymes (DNase and liberase) for one hour at 37 °C shaking at 240 rpm. The digested tissue was then macerated through a 100-µm Nytex filter and washed with Dulbecco’s modified Eagle’s medium (DMEM), and erythrocyte lysis was performed using a hypotonic ACK medium. Lysis was stopped by adding DMEM containing 10% FCS. Cells were counted using a Neubauer counting chamber and a CD90.2 MACS enrichment (Miltenyi) was performed according to the manufacturer’s instructions. Finally, cells were stained with fluorescently labelled antibodies against CD8a, CD45.1 and CD44 as well as a fixable live–dead dye. For the experiment on day 3.25 cells, single, live CD8a^+^CD45.1^+^ cells were sorted and subsequently used to prepare scRNA-seq libraries. For the retransfer experiment, single, live CD8a^+^CD45.1^+^ cells from day 4.5 after infection were sorted into PD-1^hi^ and PD-1^low^ and adoptively transferred into new host mice, which were subsequently infected with LCMV Armstrong as well. On day 15 after infection, spleens from these secondary hosts were isolated, CD8a^+^CD45.1^+^ were sorted and subsequently used to prepare scRNA-seq libraries using the BD Rhapsody system (BD Biosciences). First, sample tags were added to individual cell samples (BD Ms Single Cell Sample Multiplexing Kit, protocol 23-21340(02)). Next, single cells of the pooled samples were captured using the BD Rhapsody HT Xpress system for each experiment separately, followed by reverse transcription and cDNA amplification. Sequencing libraries were constructed using the BD Rhapsody whole transcriptome analysis approach. Quality control of libraries was done with Bioanalyzer 2100 using the High Sensitivity DNA Kit (Agilent) and Qubit 2.0 fluorometer with the Qubit 1X dsDNA HS Assay Kit (Invitrogen). Libraries were sequenced on a NovaSeq X Plus (Illumina) with paired-end 2 × 150-bp reads. Detailed information on the analysis pipelines is provided in the Supplementary Methods (scRNA-seq analysis).

### ScRNA-seq analysis of previously published data

Read counts from a previously published dataset^26^ were retrieved from the GEO database under the accession number GSE119943. Data integration was performed on the two published replicates. Subsequently, PCA, identification of nearest neighbours and Louvain^40^ clustering were performed, followed by UMAP dimensional reduction. Detailed information on these steps and further downstream analyses is provided in the Supplementary Methods (scRNA-seq analysis).

### De novo bulk RNA-seq

Total RNA was isolated using the ReliaPrep RNA Cell Miniprep System (Promega). The quality and quantity of isolated RNA were analysed with the Bioanalyzer RNA Pico Chip (Agilent). cDNA synthesis and library preparation were performed with the SMART-Seq v4 PLUS Ultra Low Input RNA kit (Takara) following the manufacturer’s protocol. Libraries were sequenced on a single-end run (1 × 100 bp, dual-index) on a NovaSeq6000 (Illumina) using SP 100 v.1.5 chemistry (Illumina).

### De novo bulk RNA-seq data analysis

Reads were processed with an adapted version of the nf-core^41^ pipeline for RNA-seq, using Nextflow^42^ (v22.04). Adapters were trimmed with Trimgalore (v.0.6.7) and trimmed reads were aligned to GRCm38 with STAR^43^ (v.2.6.1d) and quantified with Salmon^44^ (v1.5.2). Sorting and indexing of the bam files was done with SAMtools^45^ (v.1.14). PCA was performed on library-size normalized log values computed with edgeR’s (v.3.36.0) cpm function. Mean-variance trend was estimated with limma’s (v.3.50.3) voom function. Model fitting was performed with limma lmFit followed by empirical Bayes moderation, and changes with *P*_adj_ < 0.05 and |log_2_FC| > 0.58 were considered significant. Differential expression between PD-1^hi^ and PD-1^lo^ precursors for all genes from the core exhaustion signature described previously^24^ was correlated with differential expression between precursor T cells after LCMV Docile and LCMV Armstrong infections at 21 days after infection (GSE142687)^24^.

### scATAC-seq analysis of previously published data

Barcodes, peaks and matrix files from the scATAC-seq data of CD8 T cells seven days after Armstrong or clone 13 infection were downloaded from the GEO database under the accession number GSE164978. Reads were mapped to the prebuilt mm10 reference from 10X (https://cf.10xgenomics.com/supp/cell-atac/refdata-cellranger-arc-mm10-2020-A-2.0.0.tar.gz) using cellranger-atac (v.2.0.0). Data were analysed in R (v.4.1.0) using Seurat (v.4.0.3)^46^ and Signac (v.1.3.0)^47^. Features detected in a minimum of 10 cells and cells with at least 200 features were kept. Most frequently observed features were identified using FindTopFeatures with min.cutoff 10. Data were normalized using term-frequency inverse-document-frequency normalization by running RunTFIDF function before reducing dimensionality with RunSVD. Because the first LSI dimension had a strong negative correlation with sequencing depth, only dimensions 2 to 30 were considered for finding neighbours and UMAP reduction. Clusters were identified with the Leiden algorithm (Python v.3.8.10) and a resolution of 0.25. Clusters were annotated on the basis of expression scores calculated using AddModuleScore for region-based bulk ATAC-seq signatures of naive, exhausted–dysfunctional, terminally exhausted–dysfunctional and precursor exhausted–dysfunctional cells reported previously^28^. Differentially accessible features between T_pex_ and the other clusters were identified with FindMarker, restricting the search to features detected in at least 10% of either of the populations and using the logistic regression framework as a test. Differences with *P*_adj_ < 0.05 and average log_2_FC > 0.3 were considered significant. Regions were annotated to genes using the R package EnsDb.Mmusculus.v79 (v.2.99.0) and Signac’s function ClosestFeature. Features differentially accessible between T_pex_ and T_mpc_ were considered significant when *P*_adj_ < 0.05 and average log_2_FC > 0.15. Known transcription-factor-binding sites were retrieved using getMatrixSet from the package TFBSTools (v.1.32.0) and added to the object with Signac’s AddMotifs function using the genomic reference from the package BSgenome.Mmusculus.UCSC.mm10 (v.1.4.3). Transcription factors associated with the differential accessibility between T_pex_ and T_mpc_ were identified with the Signac wrapper for chromVAR (v.1.16.0) and FindMarkers applied to the chromvar assay. Tn5 insertion frequency at peaks containing FOS::JUN motifs was visualized with Signac’s Footprint function.

### Reporting summary

Further information on research design is available in the Nature Portfolio Reporting Summary linked to this article.

## Online content

Any methods, additional references, Nature Portfolio reporting summaries, source data, extended data, supplementary information, acknowledgements, peer review information; details of author contributions and competing interests; and statements of data and code availability are available at 10.1038/s41586-024-08451-4.

## Supplementary information

Supplementary Methods ScRNA-seq analysis. A detailed overview about the different pipelines used to analyse the separate scRNA-seq experiments conducted for this study. We provide information of the platform, language version and an in depth description about the different processing steps up to the clustering of single cells.

Reporting Summary

Supplementary Figure 1 Graphical summary. The image provides a graphical illustration of the main finding presented in our manuscript and summarizes the conceptual change we proposed for the development of stem-like progenitors in infections.

## Figures and Tables

**Extended Data Table 1:**
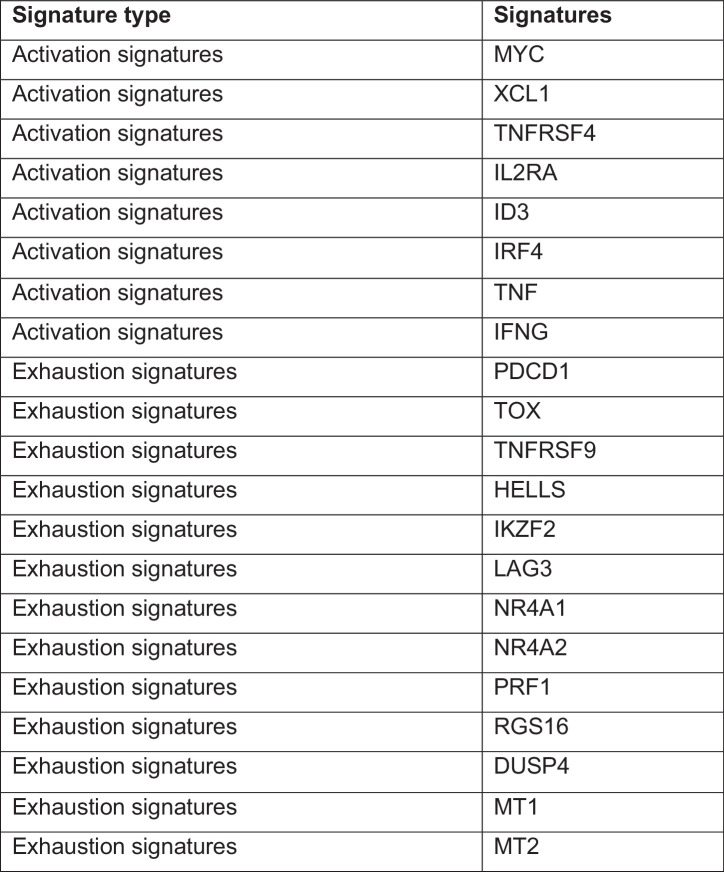
Activation and exhausted gene signatures

## Data Availability

All RNA-seq datasets have been deposited in the NCBI GEO public database under accession number GSE278807. The following published datasets available in the GEO database were reanalysed: GSE119943 (scRNA-seq), GSE142687 (bulk RNA-seq), GSE164978 (scATAC-seq). All other data supporting this study are available in the main Article and its Supplementary Information.

## Code availability

Scripts allowing the reproduction of the data will be shared by D.Z. upon reasonable request.
