# Supplementary material for: Precursors of exhausted T cells are pre-emptively formed in acute infection
Source: Nature. 2025 Jan 8;640(8059):782–92. doi: 10.1038/s41586-024-08451-4 (PMC12003159; doi:10.1038/s41586-024-08451-4)
Supplement: Supplementary file 1 — ScRNA-seq analysis. A detailed overview about the different pipelines used to analyse the separate scRNA-seq experiments conducted for this study. We provide information of the platform, language version and an in depth description about the different processing steps up to the clustering of single cells. [file 41586_2024_8451_MOESM1_ESM.pdf]

nature portfolio

https://doi.org/10.1038/s41586-024-08451-4

Supplementary information

Precursors of exhausted T cells are pre-emptively formed in acute infection

In the format provided by the authors and unedited

Antibody	Flurochrome	Company	Clone	Product #	Dilution
CD8a	Pacific Blue	Biolegend	56-6.7	100725	1:200
CD8a	BV510	Biolegend	56-6.7	100752	1:200
CD8a	AF700	Biolegend	56-6.7	100730	1:200
CD44	Pacific Blue	Biolegend	IM7	103020	1:400
CD44	BV510	Biolegend	IM7	103044	1:400
CD44	BV786	Biolegend	IM7	103059	1:400
CD44	FITC	Biolegend	IM7	103006	1:400
CD44	AF700	Biolegend	IM7	103026	1:400
CD45.1	Pacific Blue	Biolegend	A20	110722	1:200
CD45.1	AF700	Biolegend	A20	110724	1:200
CD62L	BV785	Biolegend	Mel-14	104440	1:200
CXCR5	PE	Invitrogen	SPRCL5	12-7185-82	1:30
CXCR5	PE-cF594	Biolegend	L138D7	145504	1:30
CXCR5	PE/Cy7	Invitrogen	SPRCL5	25-7185-82	1:30
IFNγ	BV711	Biolegend	XMG1.2	505836	1:200
IgG1κ	AF488	Thermo Scientific	P3.6.2.8.1	53-4714-80	1:400
KLRG1	PerCp/Cy5.5	Biolegend	2F1/KLRG1	138418	1:200
KLRG1	PE/Cy7	Biolegend	2F1/KLRG1	138416	1:200
KLRG1	PE/Cy7	Invitrogen	2F1/KLRG1	25-5893-82	1:200
Nur77	AF488	Thermo Scientific	12.14	53-5965-82	1:400
PD-1	BV421	Biolegend	29F.1A12	135221	1:200
PD-1	BV605	Biolegend	29F.1A12	135220	1:200
PD-1	PE/Cy7	Biolegend	RMP1-30	109110	1:200
TCF-1	BV421	BD	S33-966	566692	1:400
TCF-1	PE	BD	S33-966	564217	1:400
TIM3	BV605	Biolegend	RMT3-23	119721	1:200
TIM3	PE	Biolegend	RMT3-23	119704	1:200
TIM3	APC	Biolegend	RMT3-23	119706	1:200
TNF	FITC	Biolegend	MP6-XT22	506304	1:200
TOX	PE	Invitrogen	TXRX10	12-6502-82	1:400
TOX	eF660	Invitrogen	TXRX10	50-6502-82	1:400

Supplementary Table 1: List of flow cytometry antibodies used for staining.
