# Supplementary material for: Precursors of exhausted T cells are pre-emptively formed in acute infection
Source: Nature. 2025 Jan 8;640(8059):782–92. doi: 10.1038/s41586-024-08451-4 (PMC12003159; doi:10.1038/s41586-024-08451-4)
Supplement: Supplementary file 2 — Reporting Summary [file 41586_2024_8451_MOESM2_ESM.pdf]

nature portfolio

Corresponding author(s): Dietmar Zehn, Matthias Heining, Ming Wu and Carl-Philipp Hackstein

Last updated by author(s): 2024 - 10- 14

Reporting Summary

Nature Portfolio wishes to improve the reproducibility of the work that we publish. This form provides structure for consistency and transparency in reporting. For further information on Nature Portfolio policies, see our Editorial Policies and the Editorial Policy Checklist.

Statistics

For all statistical analyses, confirm that the following items are present in the figure legend, table legend, main text, or Methods section.

n/a	Confirmed	
☐	☒	The exact sample size (*n*) for each experimental group/condition, given as a discrete number and unit of measurement
☐	☒	A statement on whether measurements were taken from distinct samples or whether the same sample was measured repeatedly
☐	☒	The statistical test(s) used AND whether they are one- or two-sided<br>*Only common tests should be described solely by name; describe more complex techniques in the Methods section.*
☒	☐	A description of all covariates tested
☐	☒	A description of any assumptions or corrections, such as tests of normality and adjustment for multiple comparisons
☐	☒	A full description of the statistical parameters including central tendency (e.g. means) or other basic estimates (e.g. regression coefficient) AND variation (e.g. standard deviation) or associated estimates of uncertainty (e.g. confidence intervals)
☐	☒	For null hypothesis testing, the test statistic (e.g. *F*, *t*, *r*) with confidence intervals, effect sizes, degrees of freedom and *P* value noted<br>*Give P values as exact values whenever suitable.*
☒	☐	For Bayesian analysis, information on the choice of priors and Markov chain Monte Carlo settings
☒	☐	For hierarchical and complex designs, identification of the appropriate level for tests and full reporting of outcomes
☐	☒	Estimates of effect sizes (e.g. Cohen's *d*, Pearson's *r*), indicating how they were calculated

Our web collection on statistics for biologists contains articles on many of the points above.

Software and code

Policy information about availability of computer code

Data collection	Facs Fortessa (BD Biosciences), Facs Aria III (BD Biosciences), Cytoflex LX (Beckman Coulter), 10X genomics, Illumina NovaSeq 6000
Data analysis	FACS data – BD Bioscience FlowJo v10.09, Graph Pad Prism v.8, Microsoft Excel v16.89.1<br><br>scRNA-Seq: R v4.0.4, v4.1.0. and v4.3, python v3.8.12- a detailed overview describing the bioinformatic pipelines is included in the Supplementary Information.<br><br>RNA-seq: bcl2fastq (v2.20.0.422), R (v4.0.4, v4.1.0, v4.1.1 , v4.3.0), python (v3.8.10, v3.8.12, v3.9.6), jupyter notebook (v6.4.3), cellranger multi (v7.1.0), cellranger-atac (v2.0.0), BD Rhapsody™ Sequence Analysis Pipeline (r16), soupX (v1.6.1), scanpy (v1.8.1) , scDblFinder (v1.8.0), sctransform (v0.3.2, v0.3.3, v0.3.5), rpy2 (v3.4.5), anndata2ri (v1.1), SingleCellExperiment (v1.16.0), Seurat (v4.0.1, v4.0.3, v4.1.1, v4.3.0), SeuratDisk (v0.0.0.9021), Signac (v1.3.0), EnsDb.Mmusculus.v79 (v2.99.0), mvTCR (v0.1.1), diffxpy (v0.7.4), limma (v3.50.3, v3.56.1), velocyto (v0.17), scvelo (v0.2.4), AnnData (v0.7.6), scirpy (v0.12.2) , nextflow (v22.04), nf-core (v3.9), TFBSTools (v1.32.0), BSgenome.Mmusculus.UCSC.mm10 (v1.4.3), chromVAR (v1.16.0), TrimGalore (v0.6.7), and Cutadapt (v3.4), STAR (v2.6.1d), Salmon (v1.5.2), SAMtools45 (v1.14), edgeR (v3.36.0).

For manuscripts utilizing custom algorithms or software that are central to the research but not yet described in published literature, software must be made available to editors and reviewers. We strongly encourage code deposition in a community repository (e.g. GitHub). See the Nature Portfolio guidelines for submitting code & software for further information.

Data

Policy information about availability of data

All manuscripts must include a data availability statement. This statement should provide the following information, where applicable:

- Accession codes, unique identifiers, or web links for publicly available datasets
- A description of any restrictions on data availability
- For clinical datasets or third party data, please ensure that the statement adheres to our policy

All RNA-sequencing datasets have been deposited in the NCBI GEO public database under accession number GSE278807.
The following published data sets available in GEO database were reanalyzed: GSE119943 (scRNAseq), GSE142687 (bulk RNA-seq), GSE164978 (scATAC-seq).
The following genome references and annotation were used: refdata-cellranger-arc-mm10-2020-A-2.0.0, refdata-cellranger-vdj-GRCm38-alts-ensembl-7.0.0 (GRCm38 - gencode M23) and RhapRef_Mouse_WTA_2023-02 (GRCm38, gencode vM24).
Source data are provided with this paper. All other data supporting this study are available in the main article and Supplementary Information.

Research involving human participants, their data, or biological material

Policy information about studies with human participants or human data. See also policy information about sex, gender (identity/presentation), and sexual orientation and race, ethnicity and racism.

Reporting on sex and gender	n/a
Reporting on race, ethnicity, or other socially relevant groupings	n/a
Population characteristics	n/a
Recruitment	n/a
Ethics oversight	n/a

Note that full information on the approval of the study protocol must also be provided in the manuscript.

Field-specific reporting

Please select the one below that is the best fit for your research. If you are not sure, read the appropriate sections before making your selection.

☒ Life sciences ☐ Behavioural & social sciences ☐ Ecological, evolutionary & environmental sciences

For a reference copy of the document with all sections, see nature.com/documents/nr-reporting-summary-flat.pdf

Life sciences study design

All studies must disclose on these points even when the disclosure is negative.

Sample size	Statistical methods were not used to predetermine sample size. We chose sample sizes based on previous experience (Alfei et al., Nature, 2019, von Hoesslin et al. Science Immunology, 2022, Scherer et al. Nature Immunology 2023) and with the goal of producing statistically robust data while respecting animal welfare regulations.
Data exclusions	For TCR-scRNAseq – one sample was excluded during the FACS sorting because we were unable to obtain sufficient cell numbers owed to an unsucessfill infection, all other samples were included for sequencing and used for the subsequent analysis.<br><br>We did not exclude any samples except for rare events where the adoptive cell transfer or the infection were unsuccessful (2+1 cases in the entire manuscript).
Replication	Experiments were repeated to ensure reproducibility. Number of repeats and sample size are provided in each figure legend.
Randomization	Mice of the appropriate genotype and age were randomly assigned to the experimental groups.<br>No experiments using material from any other species were performed in this study.
Blinding	We did not perform any readouts involving subjective evaluations, such as histological analysis or clinical scoring.<br><br>Due to bio-safety regulations, we were instructed not to perform blinding in experiments involving biohazardous substances.

Reporting for specific materials, systems and methods

We require information from authors about some types of materials, experimental systems and methods used in many studies. Here, indicate whether each material, system or method listed is relevant to your study. If you are not sure if a list item applies to your research, read the appropriate section before selecting a response.

Materials & experimental systems

n/a	Involved in the study	
☐	☒	Antibodies
☐	☒	Eukaryotic cell lines
☒	☐	Palaeontology and archaeology
☐	☒	Animals and other organisms
☒	☐	Clinical data
☒	☐	Dual use research of concern
☒	☐	Plants

Methods

n/a	Involved in the study	
☒	☐	ChIP-seq
☐	☒	Flow cytometry
☒	☐	MRI-based neuroimaging

Antibodies

Antibodies used	All antibodies used, their clone and catalogue numbers as wellas dilutions used are listed in Table 2.
Validation	Antibodies were all validated by the manufacturer or previous experiments and studies (Alfei, F. et al. Nature, 2019.)

Eukaryotic cell lines

Policy information about cell lines and Sex and Gender in Research

Cell line source(s)	Vero cells, originally provided by M. J. Bevan, University of Washington<br>BHK-21 cells, originally provided by M. J. Bevan, University of Washington
Authentication	Since we used only cell-lines that we have been using in the lab for several years, we did not specifically authenticated them. However, we made sure that all used cell-lines showed the expected results and were in good condition prior to using them.
Mycoplasma contamination	Mycoplasma tests were routinely performed and were negative.
Commonly misidentified lines<br>(See ICLAC register)	To the best of our knowledge, no commonly misidentified cell lines were used in the study.

Animals and other research organisms

Policy information about studies involving animals; ARRIVE guidelines recommended for reporting animal research, and Sex and Gender in Research

Laboratory animals	C57BL/6 mice were obtained from Charles River and C57BL/6.SJL from Jackson Laboratory and both lines were maintained by intercrossing.<br>P14 TCR transgenic (provided by A. Oxenius, ETH, Zürich, Switzerland), Vb5 TCR transgenic (provided by P. Fink, University of Washington, Seattle, USA), OT-I TCR transgenic mice (purchased from Jackson laboratories) and PD-1ko P14 TCR transgenic (provided by Ping-Chih Ho (University of Lausanne, Switzerland) mice were obtained on a C57BL/6 background and maintained by crossing them to C57BL/6.SJL mice. P14 TCR transgenic Tcf7-GFP mice were generated by the Zehn lab in the past and have been published previously (Tsui et al., Nature, 2022)<br>6-13 week old male and female mice from these were used for experiments and were housed under the following conditions:<br>- light cycle is 7:00 AM on, 7:00 PM off<br>- Temperature at 22-26°C<br>- Humidity is between 30/70 g/m3
Wild animals	No samples from wild animals were used in this manuscript.
Reporting on sex	There was no discrimination of sex for these experiments. All experiments used a combination of male and female recipients and CD45.1 P14 or OT-I donors.
Field-collected samples	No field-collected samples were used in this manuscript.
Ethics oversight	Experiments performed with mice were approved by the veterinarian authorities of the 'Regierung von Oberbayern' in Germany.

Note that full information on the approval of the study protocol must also be provided in the manuscript.

Plants

Seed stocks	n/a
Novel plant genotypes	n/a
Authentication	n/a

Flow Cytometry

Plots

Confirm that:

☒ The axis labels state the marker and fluorochrome used (e.g. CD4-FITC).

☒ The axis scales are clearly visible. Include numbers along axes only for bottom left plot of group (a 'group' is an analysis of identical markers).

☒ All plots are contour plots with outliers or pseudocolor plots.

☒ A numerical value for number of cells or percentage (with statistics) is provided.

Methodology

Sample preparation	Single cell splenocyte suspensions were prepared from naive or infected mice by mashing spleen through a 100µm cell strainer followed by hypotonic ACK lysis. Total splenocytes were used for flow cytometry analysis and sorting. Following isolation, cells were incubated with Zombie NIR dye (Biolegend, 423106) and Fc-blocking reagent 2.4G2 in PBS (ThermoFisher) for 15 minutes at room temperature (RT). Next, cells were washed and resuspended in freshly prepared master mix containing fluorescent staining antibodies in fluorescence-activated cell sorting (FACS) buffer (PBS, 2% FCS, 0.1% sodium azide). When master mix contained 2 or more brilliant violet polymer dyes, Super bright staining buffer (Invitrogen SB-4401-42) was used. For tetramer or CXCR5 staining, cells were stained for one hour at room temperature. FoxP3/ Transcription factor staining buffer (Thermo Fisher 00-5532-00) was used to perform intranuclear staining following manufacturer recommended protocols. Samples were acquired on BD FACS Fortessa or Beckman Coulter CytoFlex LX instruments. For sorting: following live/dead stain and Fc block, cells were incubated with freshly prepared master mix for 20 minutes at RT, followed by wash in Magnetic-activated cell sorting buffer (PBS, 1% FCS, 2 mM EDTA). Cells were immediately run on BD FACSAria Fusion sorter. Single-stained controls were prepared using Ultracomp eBeads (Invitrogen 01-2222-42) for every experiment. Beads were treated like the cell containing samples (including fixation protocol).
Instrument	FACS data collection took place on a FACS Fortessa (BD Biosciences) or a Cytoflex LX (Beckman Coulter).<br>Cell sorting was executed on FACS Aria Fusion (BD Biosciences).
Software	FACSDiva version, FlowJo version 10.09 (BD Bioscience).
Cell population abundance	Purity of sorted cell populations was >95%.
Gating strategy	Cells from mice lacking the Tcf7-reporter construct were gated as single, lymphocyte (SSC-A vs FSC-A), time (consistent), live CD8a+, CD45.1+ or CD45.1-, CD44+ or CD44- CD62L+ CD44- . Within the CD44+ populations cell were further gated as KLRG1-. Subsequently expression of transcription factors, or within TOX+ and TOX- populations of the TCF1+ subset, the expression of Nur77 or effector molecules (IFNg and TNF) was analysed.<br>Cells expressing the Tcf7-GFP reporter were gated as single, lymphocyte (SSC-A vs FSC-A), live, CD8a+, CD45.1+ Tcf7-GFP+, KLRG1-, PD-1low or PD-1hi .<br>Gating strategies are displayed in Extended Data Figure 3.

☒ Tick this box to confirm that a figure exemplifying the gating strategy is provided in the Supplementary Information.
