# Supplementary figures and images for: Precursors of exhausted T cells are pre-emptively formed in acute infection
Source: Nature. 2025 Jan 8;640(8059):782–92. doi: 10.1038/s41586-024-08451-4 (PMC12003159; doi:10.1038/s41586-024-08451-4)

# Precursor dynamics

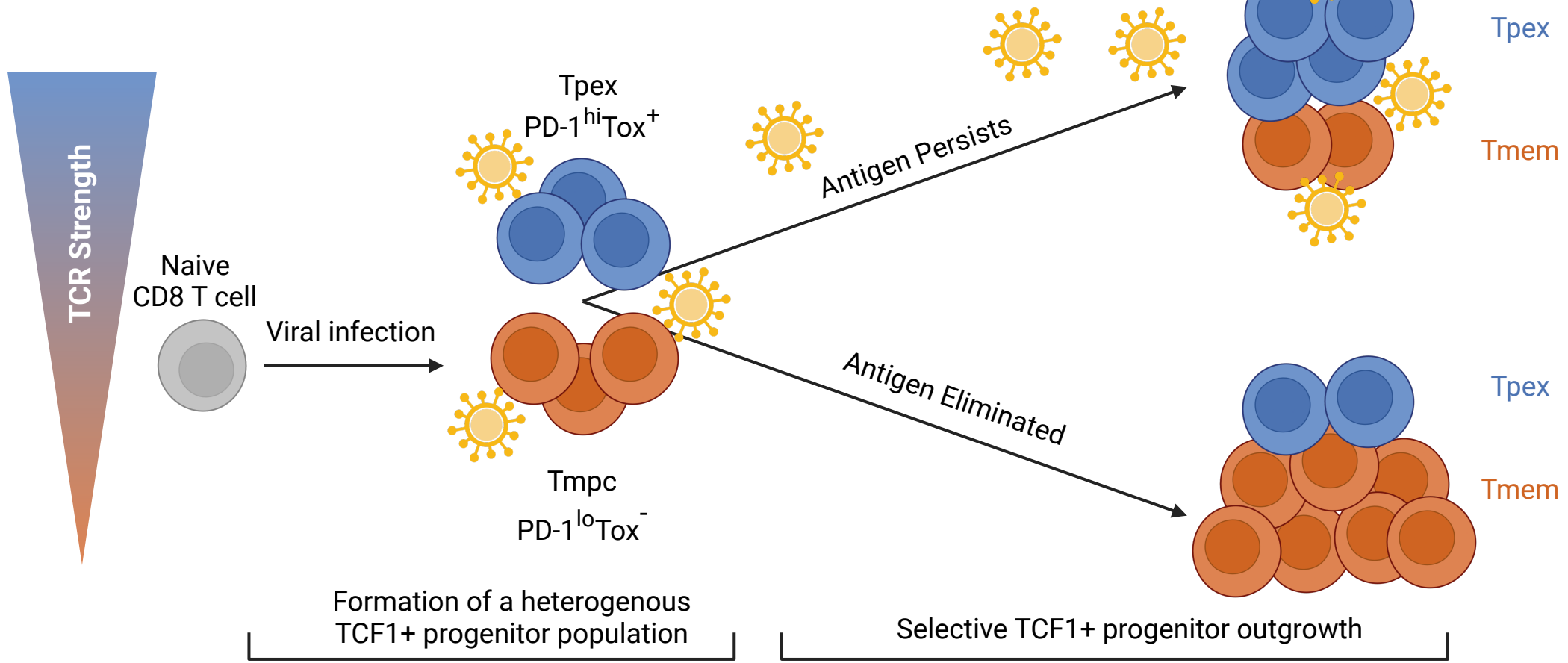

Supplement: Supplementary file 3 — Graphical summary. The image provides a graphical illustration of the main finding presented in our manuscript and summarizes the conceptual change we proposed for the development of stem-like progenitors in infections. [file 41586_2024_8451_MOESM3_ESM.pdf]
